# A Twist of Fate: The Helix–Turn–Helix Motif in *Pseudomonas aeruginosa* ExsA Can Allosterically Stabilize the Ligand-Binding Domain

**DOI:** 10.1021/acs.jcim.5c01120

**Published:** 2025-11-11

**Authors:** Prasanthi Medarametla, Jack Calum Greenhalgh, Ina Pöhner, Martin Welch, Antti Poso, Thales Kronenberger, Taufiq Rahman

**Affiliations:** 1 School of Pharmacy, Faculty of Health Sciences, 205538 University of Eastern Finland, Kuopio 70211, Finland; 2 Department of Pharmacology, 2152 University of Cambridge, Tennis Court Road, Cambridge CB2 1PD, United Kingdom; 3 Department of Biochemistry, 2152 University of Cambridge, Hopkins Building, Tennis Court Road, Downing Site, Cambridge CB2 1QW, United Kingdom; 4 Department of Pharmaceutical and Medicinal Chemistry, Institute of Pharmaceutical Sciences, Eberhard-Karls-Universität, Tuebingen, Auf der Morgenstelle 8, Tuebingen 72076, Germany; 5 Interfaculty Institute of Microbiology and Infection Medicine (IMIT), 27203 University of Tübingen;Partner-site Tübingen, German Center for Infection Research (DZIF),Elfriede-Aulhorn-Str. 6,Tübingen 72076, Germany

## Abstract

*Pseudomonas aeruginosa* is an opportunistic human pathogen. One of the most potent virulence factors in its arsenal is the type III secretion system (T3SS). This secretion apparatus injects effector toxins directly into host cells, thereby causing cytotoxicity. The expression of all components of T3SS is regulated by a master transcriptional regulator, ExsA. The inhibition of the latter should therefore lead to the suppression of *P. aeruginosa* virulence. However, to date, no drugs targeting ExsA have reached the market, and only static structural models of the protein have been generated, focusing on the C-terminal domain (CTD). Here, we used μs atomistic molecular dynamics (MD) simulations to investigate the conformational dynamics of full-length ExsA bound to DNA or DNA free, investigated as monomers or dimers. Our data show how the CTD and NTD of ExsA likely interact with one another and how ExsA binds to DNA. We also analyzed the MD trajectories to predict potential druggable pocket(s) in the structure and relevant geometry. This revealed a lipid-binding pocket within the β-sheet bundle and identified two novel potentially druggable pockets at the NTD/CTD interface, which could be used in future structure-based drug discovery campaigns. Overall, a single helix–turn–helix motif seems to drive DNA recognition in each ExsA monomer and to stabilize the putative ligand-binding domain.

## Graphical abstract

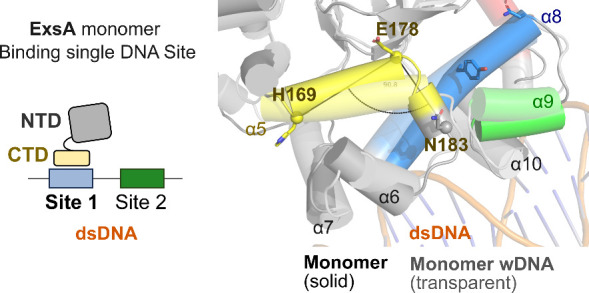

## 1 Introduction

*Pseudomonas aeruginosa* (PA) is a ubiquitous Gram-negative pathogen associated with human infections. The organism is particularly well-known to cause airway infections in people with cystic fibrosis (CF) and soft tissue infections in immunocompromised individuals.^1^ In 2017, the WHO designated *P. aeruginosa* as one of the three “critical priority pathogens”^2^ for which new antibiotics are urgently needed, yet in the intervening years, little progress has been made.^3^ However, one promising approach could be to target bacterial virulence mechanisms,^4,5^ stemming from the rationale that compromising the virulence of the pathogen will allow the host immune system a better chance of clearing the infection, thereby also synergizing the impact of conventional antibiotics. Also, resistance to such antivirulence agents, if this happens, is likely to develop less rapidly than with conventional antibiotics.^4^ One of the main virulence factors in PA is the Type III Secretion System (T3SS). The latter is essentially a needle-like “injectisome” which transports effector toxins such as ExoS, ExoT, ExoU and ExoY directly from the bacterial cytosol into the host cell. The ca. 40 open reading frames encoding the T3SS are organized into 10 transcriptional units, and the expression of all of these is controlled by the master transcriptional regulator, ExsA. The activity of ExsA is regulated by a noncanonical partner switching cascade (Figure 1A). Essentially, in the absence of induction (by host cell contact), ExsE is bound to ExsC, and ExsD is bound to ExsA. In this sequestered state, the latter remains unable to bind to DNA target sequences to activate transcription of the T3SS operons.^6^ Consequently, only a basal level of the T3SS machinery is expressed. However, this basal level of T3SS expression plays an important role since, upon host cell contact, the T3SS injectisome becomes active and ExsE is exported through the needle.^7^ This releases ExsC, which subsequently binds ExsD, the cognate antiactivator of ExsA^8^ (Figure 1 **B,C**). Thus, liberated, ExsA binds its cognate operator regions, activating expression of the T3SS operons and rapidly increasing the number of T3SS injectisomes in the cell.^9^

**Figure 1:**
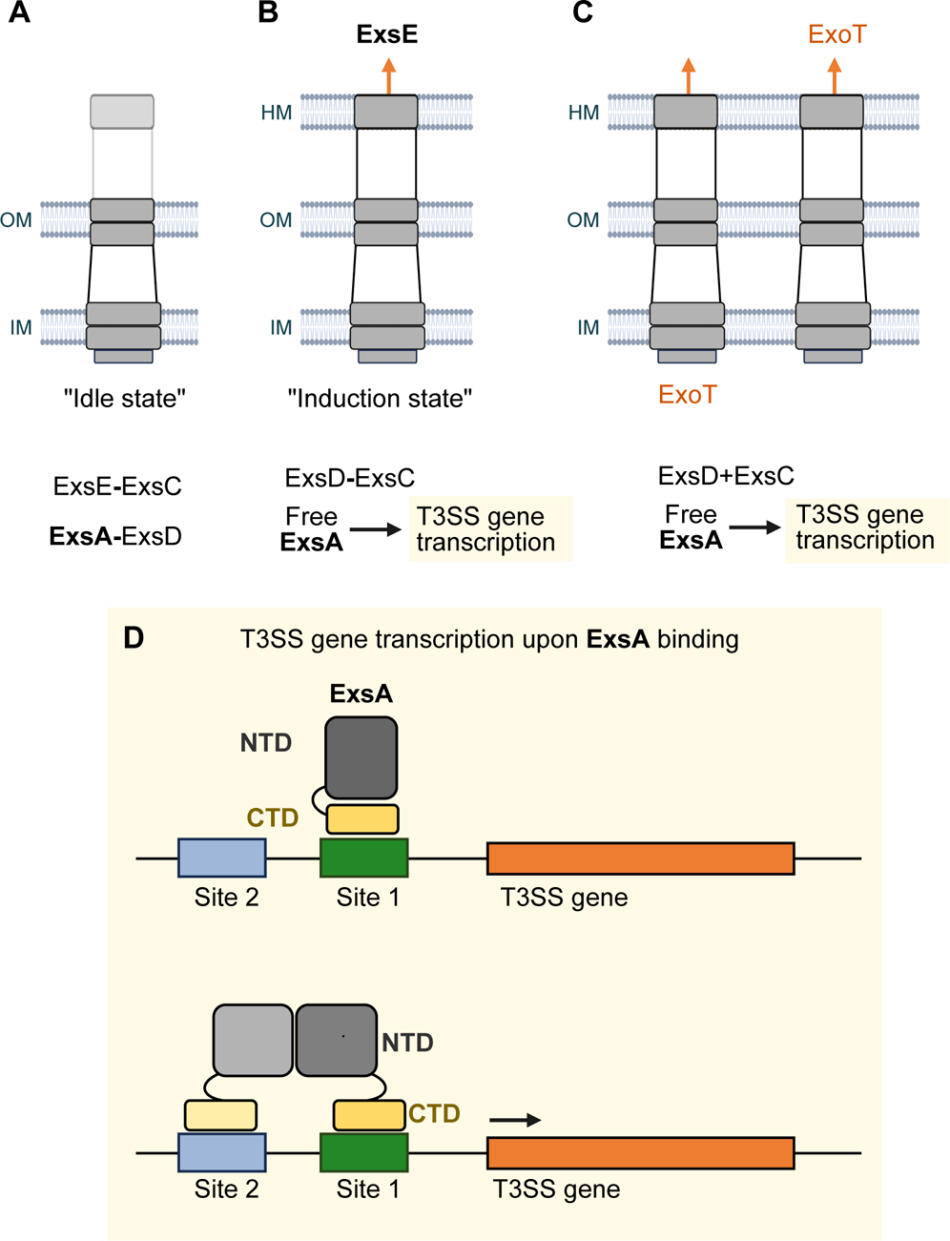
Canonical partner switching mechanism regulating ExsA activity. The layers labeled IM, OM, and HM indicate the bacterial inner membrane, outer membrane, and host membrane, respectively. In (A) the T3SS remains ‘idle’, with ExsE bound to ExsC, and ExsD bound to ExsA. Upon contact with host cells, the ExsE-ExsC complex dissociates and ExsE is secreted through the injectisome (B). This liberates ExsA to activate transcription of the T3SS operons, and consequently, export of effectors such as ExoT (C). (D) DNA binding mechanism of ExsA. The site 1 and site 2 boxes indicate the binding site for the ExsA CTDs. Dimerization is mediated by the ExsA NTD.

ExsA is a member of the AraC family of bacterial transcription factors. Members of the latter typically comprise two domains; a regulatory N-terminal domain (NTD) and a DNA-binding C-terminal domain (CTD), which recognizes specific sequences upstream of the T3SS operons. The NTD mediates ExsA dimerization and the regulatory interaction with ExsD.^8^ The NTD also contains a presumed vestigial ligand binding pocket, since no corresponding small molecule ligand has been identified to date. Free ExsA is monomeric and binds to DNA via a bipartite pair of helix-turn-helix (bi-HTH) motifs in the CTD. This binding site overlaps the −35 sequence of the promoter (Figure 1 **B,D**). The NTD then facilitates dimerization, recruiting a second ExsA monomer which binds to another, less well-conserved site further upstream.^8^ Whether the dimerization facilitates binding to this lower-affinity site is uncertain.^10^ Interruption of any stage in this process should prevent activation of the T3SS, thereby impairing PA’s virulence. For example, in a murine PA infection model, knockdown of *exsA* expression led to increased infection recovery.^11^ Furthermore, some *N*-hydroxybenzimidazoles targeted toward ExsA have been shown to disrupt binding of the CTD to DNA,^12^ although off-target inhibition of other AraC-family members remained an issue.^13^ Other potential approaches could be to target the NTD by mimicking the antiactivator ExsD, thereby blocking dimerization. However, the structure of the ExsD-ExsA complex has not yet been solved (although the structure of ExsD alone is known^14^), and significant conformational changes are likely to accompany the transition from the heterodimer (ExsA-ExsD) to the homodimer (ExsA–ExsA).

The goal of the current work was to better understand the ExsA structure and potential conformational changes that accompany its dimerization and DNA binding, with a view to identifying possible binding sites for drug-like compounds. To place this into context, hitherto very few structures have been experimentally determined for full-length AraC-family proteins, and those structures exhibit considerable diversity. The structures of the regulatory NTD and DNA-binding CTD of *Escherichia coli*’s AraC (involved in controlling arabinose utilization) have been separately determined.^15,16^ Similarly, the structure of *E. coli* Rob (involved in antibiotic resistance) has also been solved, although unlike most other AraC-family members, here the regulatory domain is at the C-terminus and the DNA-binding domain is at the N-terminus.^16^ Another structurally characterized family member is MarA (also involved in antibiotic resistance), which atypically, comprises just a DNA-binding domain.^17^ More recently, the structure of CuxR from *Sinorhizobium meliloti* has also been solved.^18^ However, CuxR is also somewhat atypical in that the second half of the bi-HTH motif is disordered, with only helices α6 to α9 being confidently distinguishable.^18^ From a structural perspective, perhaps the best-characterized family member is ToxT, a ligand-responsive protein which regulates virulence in *Vibrio cholerae*. The structure of ToxT and several mutant derivatives has been solved to high resolution.^19^ In addition, Rns structure from ETEC was solved as a dimer (PDB 6XIU), in which both subunits display distinct behaviors.^20^ Two distinct crystal-state snapshots — “open” and “closed” – are available for the ETEC. In the open form, its NTD and CTD are parted by a groove, with electron density suggesting a bound ligand that could fit decanoic acid, while the closed form leads to a tighter pocket.

The X-ray crystal structure of the ExsA NTD has been experimentally determined and is similar to the regulatory domain of the Rob protein.^21^ The ExsA NTD comprises an eight-stranded antiparallel β-sheet, which forms a cupin-like barrel. The barrel is sandwiched between two pairs of α-helices and a two-stranded antiparallel β-sheet. No experimental structure has been determined for the ExsA CTD, although sequence analyses suggest that it contains two HTH motifs connected by a rigid central helix, which fixes the relative orientation of each HTH motif. Currently, DNA binding interactions associated with the ExsA CTD have been inferred from static models based on the MarA and Rob proteins.^17,22–24^ However, to date, there have been no structural studies investigating how the NTD and CTD in full-length ExsA interact with each other, or how ExsA interacts with DNA. In the present study, we combined structural modeling and atomistic molecular dynamics (MD) simulations to investigate the conformational behavior of ExsA and its binding to DNA. Additionally, potential binding pockets were identified to aid structure-based drug design approaches targeting ExsA and thus, T3SS in PA.

## 2 Results and Discussion

### 2.1 ExsA Model Displays a Conserved AraC-Family Architecture

Because the structure of full-length ExsA has not yet been solved, models were generated using AlphaFold3.^25^ The experimentally determined ExsA NTD crystal structure (PDB 4ZUA, resolution: 2.5 Å), was used for comparison. Based on the predicted local distance difference test (pLDDT) scores,^25^ the model confidence was high (Figure 2 **A,B**) although there were a few regions of lower confidence, especially at the N- and C-termini. However, these areas are also predicted to be disordered by IUPred2A^26^ (Supporting Information, Figure S1). A disordered region, potentially involved in binding a protein partner, was detected around residue P134 using ANCHOR.^27^ This region corresponds to the unstructured linker between the helices α2 and α3 in the NTD.

**Figure 2:**
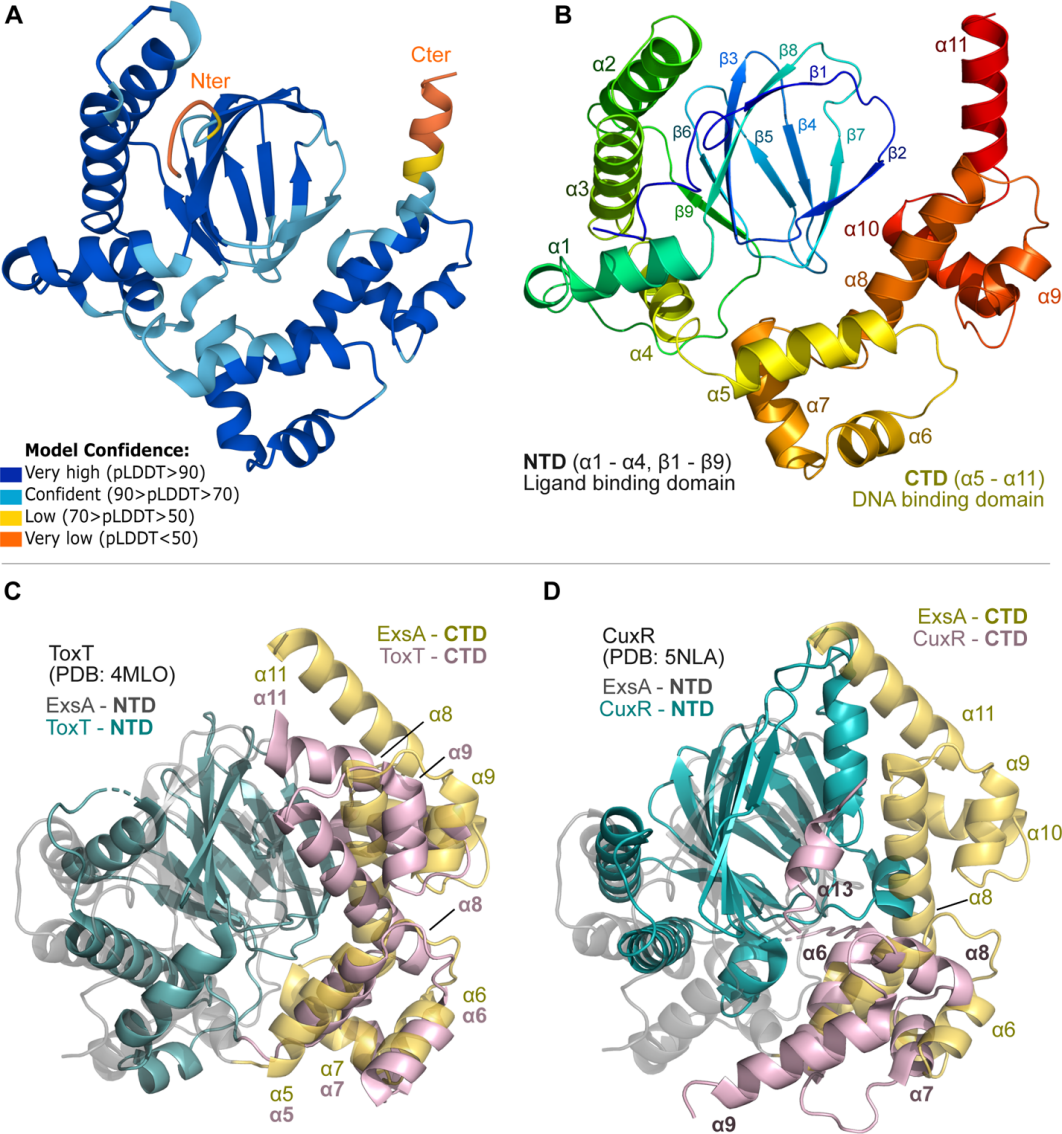
AlphaFold model of the ExsA monomer and its similarity to other full-length AraC family structures. (A) Cartoon depiction of AlphaFold-derived model of ExsA colored by the pLDDT score for each residue. (B) Secondary structure numbering of the modeled ExsA. Superimposition of the ExsA model (transparent cartoon) with the (C) ToxT and (D) CuxR crystal structures.

As ExsA belongs to the AraC family, we compared our model with experimentally determined structures of other well characterized AraC family proteins. In terms of the CTD, the ExsA model closely resembles the DNA-binding domain of AraC from *Chromobacterium violaceum* (PDB: 3OIO, Supporting Information, Figure S2A) except for a shorter α11. The DNA-bound MarA structure (PDB 1BL0, Figure S2B) was also similar, though it displayed additional small conformational changes in α9 and α10 and had a largely unfolded α11. However, when we compared the predicted full-length ExsA with ToxT (PDB 4MLO^19^) and CuxR (PDB 5NLA^18^), larger conformational rearrangements were apparent, especially in the CTD (Figure 2 **C,D**). These differences can be a result of crystallization conditions or simply unique CTD-NTD architectures explored by the different species. However, these data highlights how dynamic proteins from the AraC-family can be and, based on that, we hypothesized that ExsA’s conformation would also be diverse. ExsA is likely to be dynamic, raising the question of how DNA binding and dimerization affect its structure.

The structure of another full-length AraC-member named Rob has been solved in complex with DNA (PDB 1D5Y). However, the Rob DNA binding domain is in the NTD. By contrast, in MarA, the DNA binding domain is located in the CTD, as it is in ExsA. Also, and similar to MarA, the CTD of ExsA contains a bipartite HTH DNA-binding motif.^8^ Therefore, to investigate whether binding to double-stranded DNA (dsDNA) might alter the conformational relationship between the NTD and CTD, we generated a model of the ExsA monomer (M) in complex with nonspecific DNA (M_DNA_), and in complex with the experimentally verified ExsA operator regions derived from the P_exoT_ promotor,^28^ at site 1 (M_DNA_s1) and site 2 (M_DNA_s2).

The CTD with bound nonspecific and specific DNA were modeled based on the MarA-DNA structure (PDB 1BL0,^17^ resolution: 2.3 Å). This model explains how DNA binding influences the conformation of monomeric ExsA without introducing the confounding variable of NTD dimerization.^6,8^ Previous studies^28,29^ suggest that ExsA binds first to DNA at site 1 as a monomer and subsequently recruits another ExsA monomer at site 2, with the NTD facilitating the dimerization. To explore this dimerization, three dimeric models were generated using the AlphaFold3 (AF3); dimer without DNA (D_apo_), and two types of dimers. One dimer binds to both DNA sites (Type 1, D_DNA_s1), and another dimer with major occupation of DNA site 1 (Type 2, D_DNA_s2), with the CTDs discounted from the NTDs to allow interaction with minimal DNA bending. These dimeric models are consistent with the Rns dimer (PDB 6XIU), in which both DNA-binding domains are oriented on ∼180° allowing, in that case, a DNA looping and promoter binding.^20^ This is especially relevant as Rns targets promoters with sites ∼40 bp apart, which is longer than the ExsA’s.

Finally, MD simulations were conducted on all seven systems (Figure 3) to investigate the protein conformational changes that accompany DNA binding, providing mechanistic insights into transcriptional regulation by ExsA. In the following sections, we discuss how the identified structural rearrangements facilitate DNA recognition and dimerization.

**Figure 3:**
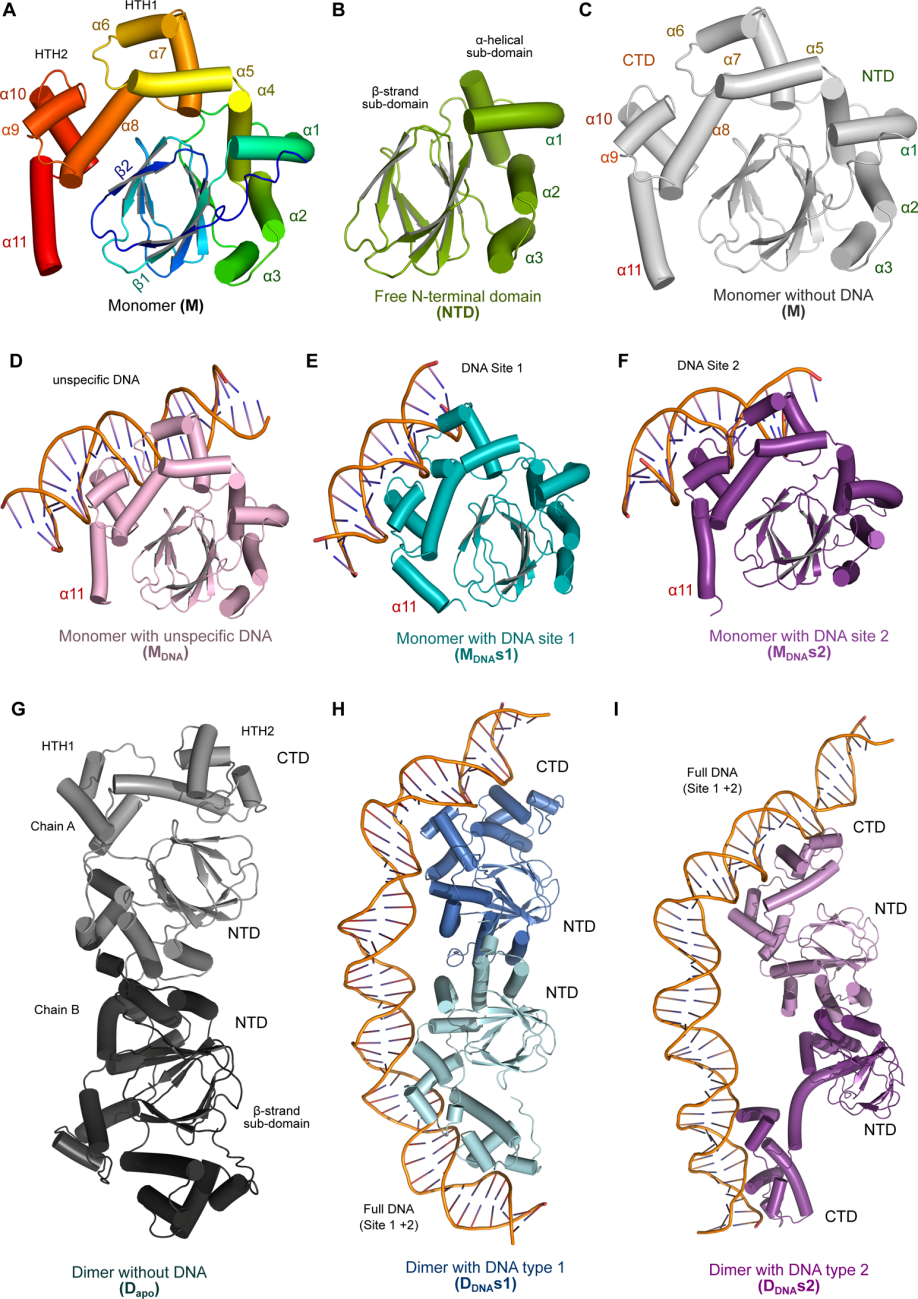
Overview of the modeled systems. (A) ExsA monomer colored in rainbow by the residue number, model generated using AlphaFold. (B) ExsA N-terminal domain (NTD; PDB:4ZUA, green). (C) Full length monomeric ExsA model generated using AlphaFold. (D) ExsA monomer bound to nonspecific DNA, derived from MarA crystal structure (PDB:1BL0); (E) ExsA monomer bound to the P_exoT_ promotor site 1 (F) ExsA monomer bound to the P_exoT_ promotor site 2. (G) Dimeric ExsA apo structure without DNA. (H) Dimeric ExsA bound to the P_exoT_ promotor (Type 1 dimer). (I) Dimeric ExsA bound to the P_exoT_ promotor (Type 2 dimer).

### 2.2 Conformational Flexibility of the NTD and Its Allosteric Interplay with the CTD

The MD simulation trajectories of the regulatory ExsA N-terminal domain (NTD), the full-length ExsA monomer with- and without bound DNA (namely: M, M_DNA_, M_DNA_s1, M_DNA_s2), and of the full-length ExsA dimer with- and without bound DNA (Dapo, D_DNA_s1 and D_DNA_s2) were used to examine the conformational flexibility of the protein (Figure 3). RMSD (root-mean-square deviation) analyses showed that all the systems had equilibrated within the corresponding simulation time scale (Supporting Information, Figure S3). At the level of individual residues, the highest RMSF (root-mean-square fluctuation; a measure of the displacement of side chains) was around the N- and C-termini. Reassuringly, the pattern of RMSF variation was very similar in both the isolated NTD (on average ∼2 Å) and the NTD of the full-length monomeric ExsA structure (also on average ∼2 Å) indicating the reliability of this structure (Supporting Information, Figures S4). However, the fluctuations in the CTD are around 5 Å in the monomer and <4 Å in the DNA-bound monomeric systems (M_DNA_, M_DNA_s1, and M_DNA_s2). Furthermore, fluctuations of the NTD and CTD in the full-length dimer systems are higher >6 Å, when compared with the isolated NTD and monomeric systems (around 5 Å). Interestingly, we noted that upon binding of either specific or unspecific DNA to the CTD, many regions in the NTD of the protein appeared to rigidify, as reflected by lower RMSF values (Supporting Information, Figures S4).

To further quantify conformational flexibility in the NTD, intradomain distances were measured over the simulation time using key marker residues from the alpha helical subdomain of the NTD (I78, L80, S81, L128, C139 and L165) and from the beta-strand-rich subdomain of the NTD (E23, R25, K28, Figure 4 **A,B**). The distances between residues in the alpha helical subdomain remained similar across all the examined systems except in the isolated NTD and in the free full-length ExsA monomer (M, Table S1). By contrast, the distances between R25 (β2) and K28 (β-strand subdomain) to W77 (β8) exhibited distinct patterns in M_DNA_s1 and M_DNA_s2 compared with all the other systems (Figure 4 **B,** Table S1). Briefly, the R25–W77 Cα distance in the isolated NTD, full-length M, and M_DNA_ systems is on average 12 Å, and remains at ∼ 10 Å in the dimeric systems. However, this distance greatly increases in M_DNA_s1 (to 24 Å) and M_DNA_s2 (30 Å). This increase in distance likely results from the recognition of specific DNA sequences by CTD, potentially enhancing the flexibility of this region. The β8 strand containing the marker residue (W77) is located adjacent to the α1 helix, which is connected to β9 by a highly flexible loop.

**Figure 4:**
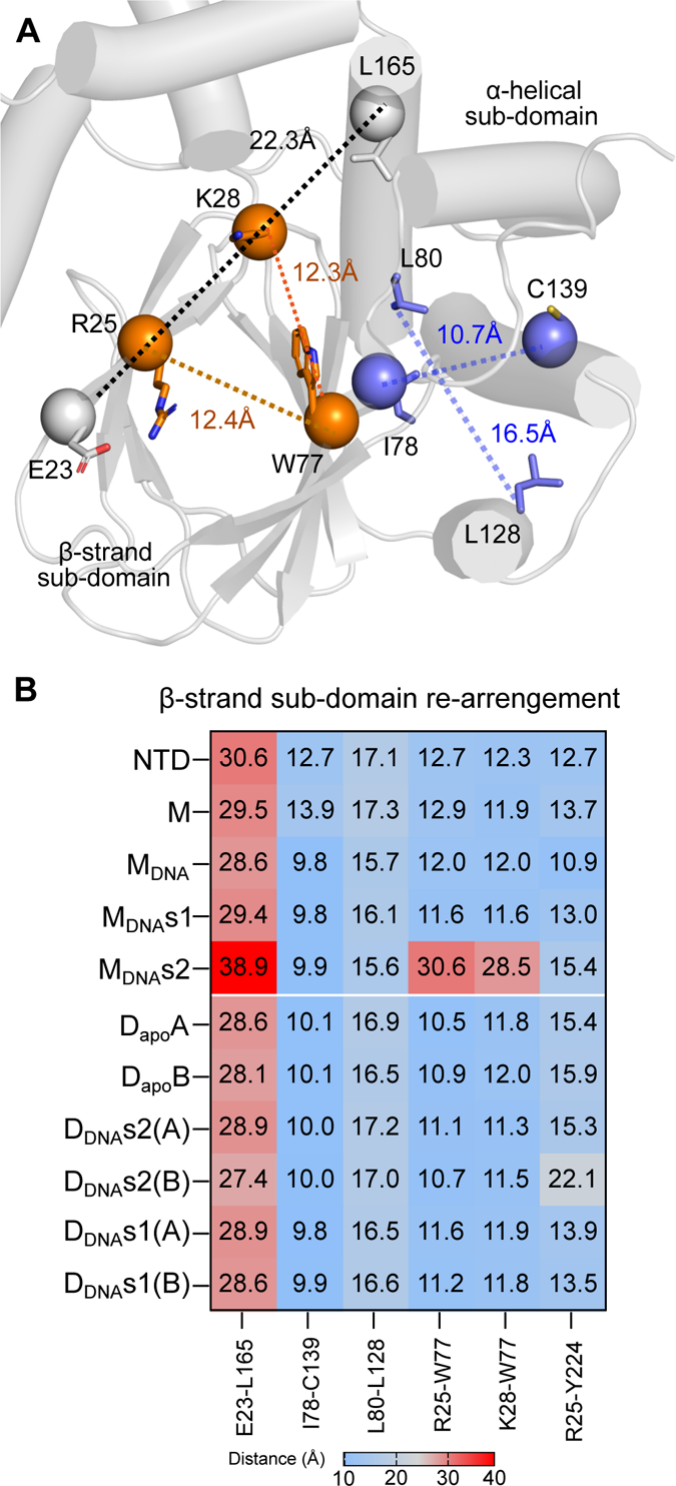
Quantifying the conformational flexibility in ExsA’s NTD. (A) Distances between Cα atoms of key marker residues in the NTD that were used to monitor conformational change. (B) Heatmap with the mean distance between the key marker residues in the NTD during the simulation.

The movements of this loop were well captured in the extreme motions observed in our principal component(s) analysis (below and Figure 5). We hypothesize that the loop might be responsible for translating the changes from β8 to α1, and to the dimerization interface. This suggests that DNA binding to the CTD influences the β-strand subdomain of the NTD, and that the conformations of the NTD and CTD are interdependent. We conclude that changes in one of these domains can allosterically influence the other.

**Figure 5:**
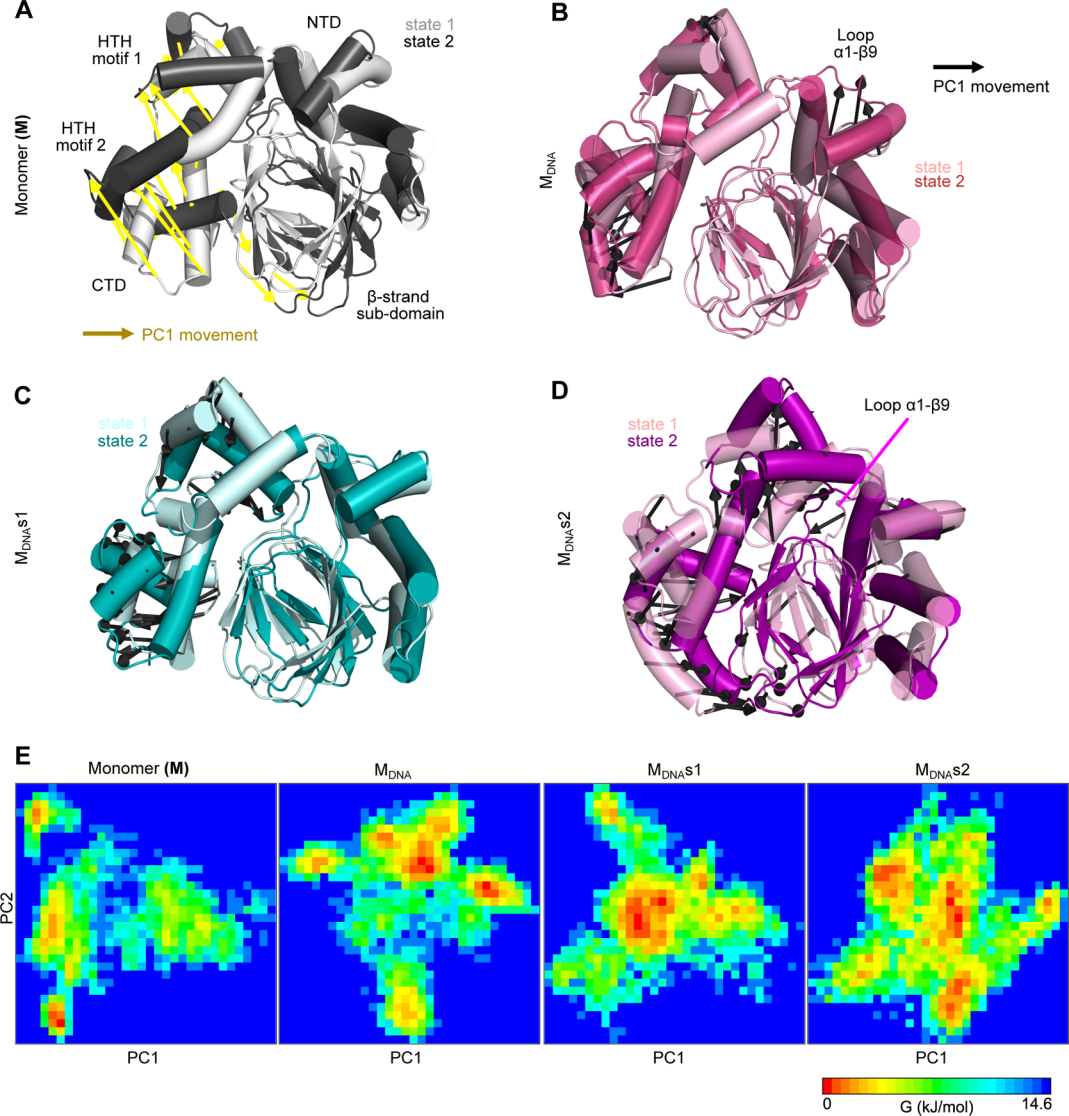
Conformational rearrangements accompanying DNA binding to the ExsA monomer. The figure shows the conformational changes observed in ExsA monomers during the MD simulation. State 1 represents the starting conformation, whereas state 2 corresponds to the conformation observed during the simulation. PC1-associated movements are indicated in black except in the Monomer (M) which are indicated in yellow. Arrows indicate the direction of motion. Conformational changes identified through principal component(s) analysis (PCA) of (A) Monomer (B) ExsA bound to nonspecific DNA (C) ExsA bound to the P_exoT_ promotor site 1 (D) ExsA bound to the P_exoT_ promotor site 2. The loop connecting the α1 and β9 is highlighted in panels B and D. (E) Gibbs free energy landscape (FEL) analysis which derives the relative free energy based on the principal components (PC1 and PC2) depicted in kJ/mol. The red indicates low energy and blue indicates high energy. The free energy value zero indicates the lowest energy state or conformation. Representative structures of the lowest energy states (derived from the red colored bins associated in the graph) are available in Figure S5.

### 2.3 Insights into the Dynamics of ExsA DNA Binding and Dimerization

We further investigated the most relevant motions for each simulated system using principal component(s) analysis (PCA). The extreme motions associated with the first principal component (PC1) in the free monomer simulations corroborate the high flexibility in the β-strand subdomain of the NTD and the whole CTD (Figure 5 and Supporting Information, Figure S5 and S6). However, the NTD is stabilized upon DNA binding, and displays minimal movements independent of whether the bound DNA sequence is specific or nonspecific (Figure 5 **B-D**). Conversely, the CTD displayed large motions in the DNA-bound systems (i.e., M_DNA_s1 and M_DNA_s2), suggesting that the binding of either site 1 DNA or site 2 DNA to the helix-turn-helix (HTH) motif in the CTD induces significant conformational changes relative to the initial model. These DNA-induced conformational changes in CTD were associated with the ExsA monomeric systems.

Therefore, we investigated how dimerization might impact on the conformation and dynamics of the CTD and NTD. Along our simulation time, and in the absence of bound DNA, the free ExsA dimer reorganizes by slightly changing HTHs conformations. This is consistent with the proposed model that an ExsA monomer first binds to site 1 and then recruits a second monomer to site 2 to form the dimer.^8,30^ By contrast, the structural rearrangements associated with PC1 in the Type 1 DNA-bound dimer (D_DNA_s1) were much smaller (Figure 6 **A,C**), while small movements concerted CTD and NTD rearrangements are observed in Type 2 to accommodate the longer bound DNA (Figure 6 **B,C**). These data suggest that, as in monomeric systems, DNA binding contributes to structural stabilization. This is also evident from the Gibbs free energy landscape (FEL, Figures 5 **E** and[Fig fig6] **C** and Figures S5 and S6) analysis which derives the relative free energy based on the principal components (PC1 and PC2). The monomer (Figure 5E) and Type 2 dimer (Figure 6C) display multiple energy basins, indicating conformational diversity and instability of the systems. In contrast, the DNA-bound monomers M_DNA_s1 (Figure 5E) exhibit a single well-defined low-energy basin, implying that DNA binding stabilizes distinct conformational states. The M_DNA_s2 displayed multiple dispersed low-energy basins reflecting the conformational diversity in which different states can interconvert. Similarly, the Type 1 DNA-bound dimer (Figure 6C) shows an increase in low-energy basins (∼11 kJ/mol) compared to those forms, reflecting greater stability and structural adaptation during DNA binding. Lastly, the DNA-free dimer has a single relevant low-energy basin (∼14 kJ/mol), distinct from the DNA-bound structures, which is consistent with an incompetent conformation.

**Figure 6:**
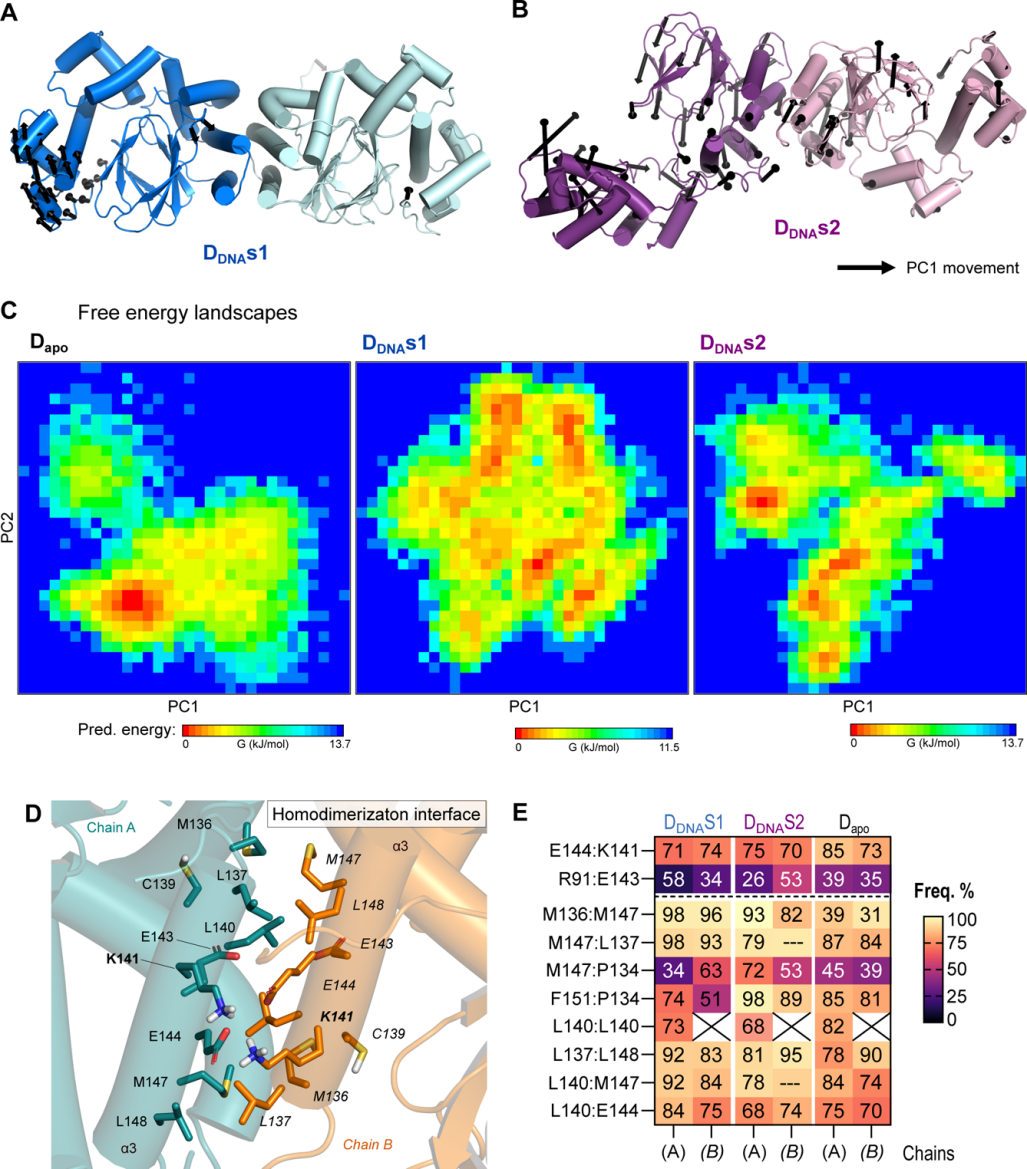
Conformational rearrangements accompanying DNA binding to ExsA dimers. The figure shows the conformational rearrangements observed in ExsA dimers during the MD simulation. Movements associated with the first principal component (PC1) are highlighted in black, with arrows indicating the direction of motion. Conformational changes identified through principal component(s) analysis (PCA) of ExsA dimer bound to the P_exoT_ promotor Type 1 (D_DNA_s1, A) and Type 2 (D_DNA_s2, B). (C) Gibbs free energy landscape (FEL) analysis which derives the relative free energy based on the principal components (PC1 and PC2) depicted in kJ/mol. The red indicates low energy and blue indicates high energy. Zero indicates the lowest energy state or conformation. Representative structures of the lowest energy states (derived from the red colored bins associated in the graph) are available in Figure S5. (D,E) Quantifying interactions between residues at the dimerization interface. (D) Interface between monomers in the ExsA dimer, highlighting the main residues from α3 involved in stabilizing the dimer. (E) Heatmap showing the frequency of interactions among the residues involved in dimerization.

We then investigated the changes in the α-helical subdomain of the NTD (associated with dimerization). This was done by studying the interaction and distances of key residues at the interface between the interacting monomers in the dimer (Figure 6 **D,E**). These analyses highlighted the relevance of helix α3 in dimerization. For both DNA-bound proposed dimers, residues from α3, namely P134, M136, L137, L140, M147 and F151 formed stable hydrophobic interactions, with frequencies higher than 70% across the analyzed simulation time (Supporting Information **Appendix datasheet**). By contrast, the free dimer conformation (D_apo_) is mostly (>50%) sustained by the interactions between M147 and M136 or M137 and further loosely hydrophobic contacts.

Consistent with this result, residues L137, C139, L140, and L148 have been previously reported in the literature as playing critical roles in ExsA self-association and activity.^10^ The leucine triad of L137, L140, and L148 can be seen in Figure 6 **D,E**. Similarly, earlier mutagenesis studies also implicated K141 and E144 as being important for ExsA transcriptional activity.^10^ Our data confirm this, showing that a stable salt bridge forms between K141 and E144 in both the DNA bound dimers (across around ∼ 75% of the MD simulation window) and in the apo dimer (across 80% of the MD simulation window).

### 2.4 Movements between ExsA Domains Change the Conformation of the DNA-Binding Sites

We wanted to better understand how DNA binding leads to greater conformational stability in ExsA, and in particular, how interactions between the NTD and CTD might be involved. To do this, we measured the distance between Cα atoms of R25 (a stable site on the NTD) and Y224 (part of the HTH motif on the CTD) in the DNA-bound and DNA-free ExsA structures over the course of the simulation (Figure 7A). The distance between these residues is smaller in M_DNA_ (10.8 Å) and M_DNA_s1 (8.7 Å) compared with that in the free monomer (13.7 Å). However, this distance increased to 15 Å in M_DNA_s2 and to a larger 13–22.1 Å in the dimeric systems (Figure 7B), with the Type 1 dimer seeming the most stable and monomeric-like, while Type 2 uncouples the CTD from the NTDs’ movements. In the M_DNA_s1 model, the binding of site 1 DNA to the CTD caused movements in the HTH motif. This, in turn, led to a decrease in the distance between R25 (on strand β2 in the NTD) and Y224 (on helix α8 in the CTD).

**Figure 7:**
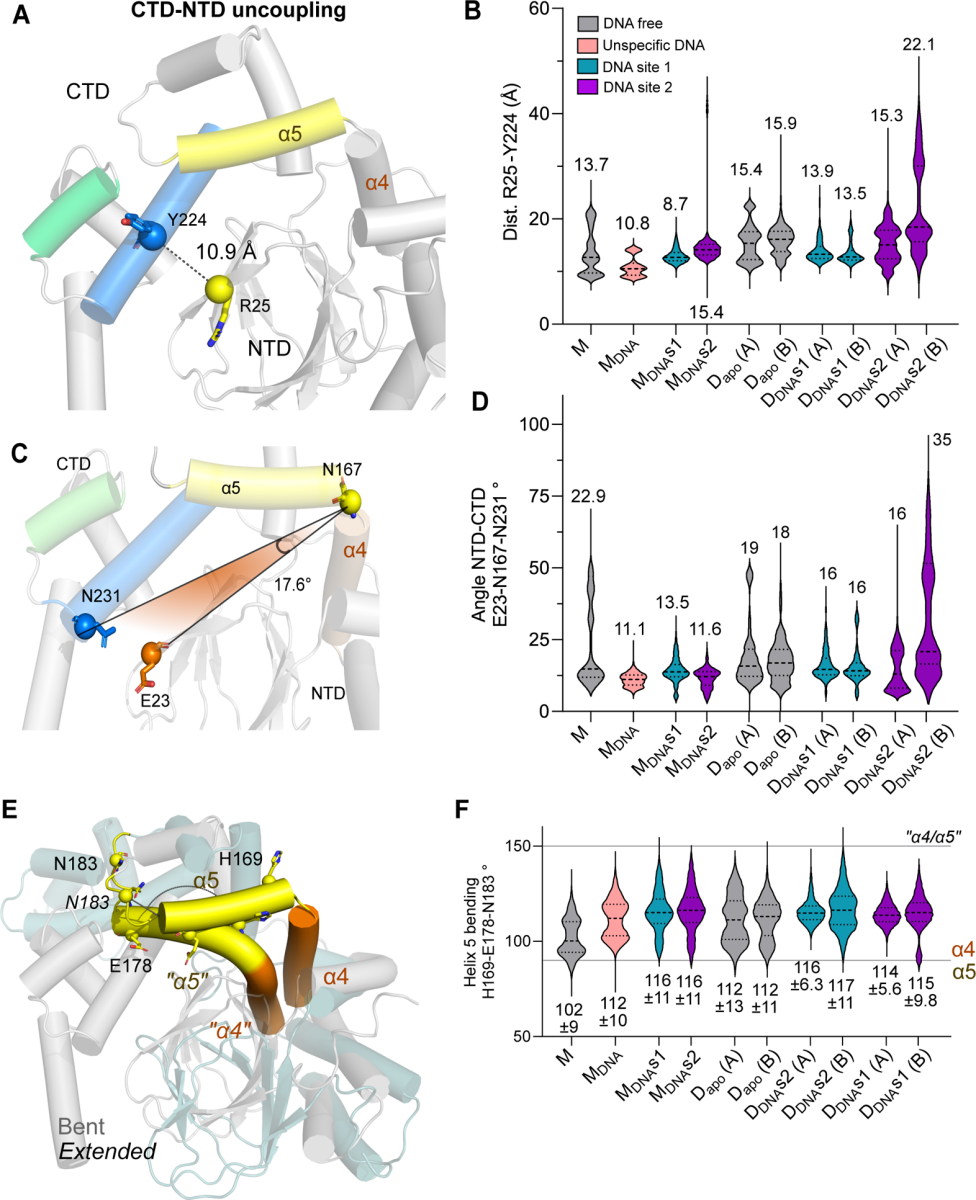
NTD and CTD of ExsA are conformationally flexible relative to each other. (A) Cartoon representation showing the distance between R25 on the NTD and Y224 on the CTD. Distances were calculated based on the Cα atoms of these amino acids. (B) Cumulative distribution of the R25-Y224 distances over the course of the MD simulation. (C) Cartoon representation of the interdomain angle (∠), defined by the Cα atoms in residues E23 on the NTD, Q167 in the hinge region, and Q231 on the CTD. (D) Angle variation (angle between Cα of E23-Q167-Q231) for the indicated structural systems in presence and absence of bound DNA during the MD simulation. For all measurements, the mean values of ∠ are given. (E) Configuration of helices α4 and α5 in the HTH motif of the ExsA monomer and dimer. Note that in the monomer they exist as distinct, separate helices. (F) Cumulative distribution of the angle between helices α4 and α5 along the simulation.

Next, and to further quantify possible large-scale movements between the domains, we measured the angle (∠) in the vertex formed between the Cα of residues E23 (located on the NTD), N167 (located in the hinge region between the NTD and CTD), and N231 (located on the CTD, Figure 7C). In the initial static model of DNA-free monomeric ExsA, the angle was a rather tight ∠17.6°, although during the simulation it widened to a mean value of ∠22.9° (Figure 7C). This suggests that in the absence of DNA, the configuration of the NTD relative to the CTD in the monomer is rather flexible. However, even in the presence of bound DNA (M_DNA_) the mean angle (∠11°) between the two domains did not change much. Much larger differences were seen when domain orientations were measured in the dimeric systems (Figure 7D). Overall, these data suggest that DNA binding to ExsA leads to a significant “tightening up” of the structure, both at the level of individual domains (the NTD and CTD), and in terms of interdomain dynamics. This tightening up is also somewhat asymmetric, since the two chains in each dimer behave slightly differently (Figure 7D). Corroborating these findings, center of mass variations between the NTD to CTD displays a similar trend; here again, the binding of DNA stabilizes both the monomeric and dimeric forms of the protein (*cf*. the apo form, Figure S7A).

The full-length ExsA binds to DNA via the HTH motifs in its CTD. The HTH motif is bipartite, comprising HTH1 (α6 and α7) and HTH2 (α9 and α10). The HTH motifs themselves are relatively rigid, but are supported by a pair of helices, α5 and α8 (depicted in yellow and blue, respectively, in Figure 7A) that act as a “structural spine”. To evaluate how the conformation of the CTD changes upon binding DNA, we measured the angle (∠) formed between Cα atoms of three residues in α5: H169, E178, N183 (Figure 7 **E,F**) in presence and absence of bound DNA. In the initial static model structure, these three residues are essentially linearly aligned in α5. However, our simulations revealed that in the M_DNA_ systems, helix α5 bends over a wide range of angles, and can transform to two helices, leading to a distinctly bimodal distribution of angles (Figure 7F). In the absence of DNA, ∠ adopted a wide range of values, with an average of 102° ± 9, whereas when bound to specific DNA fragments, the angle became more obtuse (116° ± 5.6). In stark contrast, in the dimeric systems angle variations are unimodal, where α4 and α5 are almost linearly disposed to each other (with ∠∼120°), which helps to orient the HTH motif to favor DNA binding (Figure 7 **E,F**). In view of this, we hypothesized that this transition of α4 and α5 from separate helices to a single helix may be linked with the CTD reconfiguration that accompanies dimerization (Supporting Information, Figure S7A).

This prompted us to further investigate the CTD interaction with DNA, by measuring the distance between the CTD (residues 192–268) center of mass and the bound DNA fragments (Supporting Information, Figure S7B). In simulations for the monomeric systems, the mean distance between the CTD and the DNA was shorter for the P_exoT_ DNA fragments (M_DNA_s1: ∼ 16 Å and M_DNA_s2: ∼ 17 Å), than it was for the nonspecific DNA (M_DNA_: ∼ 20 Å). Similar results were obtained for the dimeric systems too, indicating that DNA binding to the CTD stabilizes the protein in both the monomeric and dimeric forms. Closer inspection of the data revealed that although both HTH motifs remain near the DNA, the α5 rearrangements mentioned above lead to withdrawal of α9 (in HTH2) from this interaction over ∼ 30% of the analyzed simulation time, accounting for its distinctly bimodal distance relationship with the target DNA. Our data therefore indicate that movements in α5 (which forms part of the scaffold holding HTH1 in place) also impact the HTH2 conformation (Figure 7 **E,F**). This is at most pronounced-on DNA-free monomers or monomers bound to unspecific DNA fragments (Figure 7E), suggesting larger α5 bending.

### 2.5 Mutants on the DNA-Binding Interface Can Lead to NTD-CTD Uncoupling

The conformation of the HTHs is relevant to allow DNA binding. Indeed, specific residues in the HTH2 motif, such as Q253, R256 and R257 seem to retain stable polar interactions with phosphate groups on the DNA backbone, whereas Y250 (α10) engages in p-mediated interactions with the DNA bases. Commensurate with this, mutation of Y250 and R257 to alanine led to decreased DNA binding activity.^29,30^ Additionally, we identified hydrogen bonds between the DNA and the side chains of residues including S218 (α8) and T199 (α7), as well as salt-bridges with residues K202 (α10), and K186 (α6) in most of the systems studied here. We also identified intermittent interactions (>30% of the simulation time) between site 1 DNA and residues L187 and S188 in the HTH1 motif of D_DNA_s1. However, these interactions were observed for less than 10% of the MD simulation time in the other monomeric and dimeric systems.

Genetic data using the P_exoT_ promoter indicates that residues L198 and T199 in the HTH1 contact the guanine in the GnC sequence (DNA site 1). Also, HTH2’s residues Q248, Y250, T252, and R257 contribute to the recognition of the TGnnA sequence.^31^ We chose to further investigate these residues’ role on the protein conformation and DNA binding. For that we generated alanine mutants for the entire HTH1 (the so-called 3Ala-HTH1 containing L198, T199 and K202) and another additionally mutating HTH2 (including the residues Y250, T252 and R257, 3Ala-HTH1/3Ala-HTH2) in comparison to the WT M_DNA_S1. These mutants were chosen from literature^31^ as the most relevant in genetic experiments, in order to maximize its effects. Both mutants largely disrupted the ExsA monomeric conformation with the most relevant differences in the CTD-NTD angle values of 3Ala-HTH2 (∼10° larger, Figure S8), being comparable to monomeric simulations without DNA (i.e., the most flexible). In confirmation to the observed experiments, the distance between the HTHs and DNA fragments is the most divergent in the3Ala-HTH1/3Ala-HTH2 mutants (Figure S8) significantly increases by 17 Å.

The impact of single mutations was then evaluated using metadynamics. We opted for two relevant collective variables (CVs) for evaluating the CTD-NTD uncoupling: (i) the distance between R25 and Y224 (mapped on increments of 0.05 Å, up to 10 Å) and (ii) angle between E23–N167-N231 (mapped on increments of 2.5°). The CV R25-Y244 generated a clear energy minimum for WT M_DNA_s1 around ∼ 7–8 Å, as well as for the L198A, K202A and Y250A mutants (Figure S9). Alternatively bimodal distributions, meaning with a second energy minimum (R25-Y244 ∼ 5 Å), support a scenario where the space between CTD-NTD collapses for R257A and T199A mutations. This explains why those two residues are so relevant for ExsA activity,^31^ as we propose that they play a role not only stabilizing the DNA but also on maintaining the protein conformation. Most of the simulations varying the E23–N167-N231 angle as CV were highly unstable (Figure S9), with energy minima around ∼ 90°, highlighting their relevance for the overall conformational change. We hypothesize that longer time scale unbiased simulations or better selected collective variables would be relevant to fully grasp the mutation effects.

Previous simulations with MarA indicate binding at two adjacent recognition sites on the DNA sequence.^24^ By contrast, Rob appears to primarily bind to only one site in its bipartite DNA recognition sequence. This is consistent with previous observations that DNA binding by AraC-family proteins is mainly driven by interactions with one recognition sequence, even if the overall recognition motif is bipartite.^22,24^ In this regard, we note that the ExsA’s DNA binding sites are centered −44 and −65 nucleotides upstream of its target genes’ transcriptional start site: a separation of 21 bp. Given the DNA binding sites of MarA and Rob are separated by just seven nucleotides, it seems very likely that each monomer of ExsA binds independently to these sites. Interestingly, Schleif^32,33^ postulates that since AraC proteins interact with more than 40 bp, they must be partially unfolded in the DNA’s absence to allow their reversible interaction with a reasonable binding energy. This is consistent with the large conformational rearrangements we observed in our type 2 dimer simulations. However, it does not agree with dimeric crystal structures of ETEC Rns, that show little to none CTD-NTD uncoupling, which is consistent with our type 1 dimer (D_DNA_s1). Therefore, we propose that the type 1 dimers to be more biologically relevant.

### 2.6 Potential Druggable Binding Pockets and Hotspots on ExsA

To aid the drug design efforts toward ExsA, we applied a combination of structure-based tools to investigate possible druggable binding pockets. Combining predictions from different pocket detection tools and strategies can potentially point to relevant and druggable binding pockets. These consensus predictions from SiteMap, PeSTo, PocketMiner, APOP and PASSer identified four possible pockets in the full-length monomeric ExsA; (i) Orthosteric Site (ii) Dimerization Interface (iii) HTH1-β barrel pocket and (iv) α12/α13 HTH pocket (see Table 1, for definition, and Figure 8 **A-E**).

**Table 1:** Pocket Residues as Defined by Visual Analysis of the Pocket Prediction Results and Literature^a^

pocket	residues	**predict**ion tool and frames
orthosteric	W17, I19, T21, E23, R25, V26, N27, K28, E29, Y33, L40, V42, L57, V59, Y64, V66, T68, K69, I75, W77, I109, N231	APOP (frames 1, 2)
		SiteMap (frames 1, 2)
dimerization	M1, K5, S6, L7, G8, R9, K10, Q11, I12, K28, E29, E30, R60, R61, W77, P79, L80, S81, A82, F84, L85, Q86, H133, P135, M136, C139, I142, L165, R168, E171, Q174	APOP (frames 1, 2, 5)
		SiteMap (frame 2)
HTH1-β barrel	V26, N27, K28, D44, I45, D46, S47, F49, R60, R61, G62, S63, Y64, P105, V106, P107, G108, I109, Q164, V170, L173, Q174, L175, M177, E178, V208, Y209, G210, V211, S212, A215, W216, E219, R220, L223, R258, N278	APOP (frames 1, 2, 3, 5)
α12/α13 HTH	P20, T21, F22, E23, Y24, R25, T41, Q43, D46, T48, V65, K69, L223, H226, Q227, L230, N231, F245, Y250, Q253, S254, R256, R257, R258, F259, S266, E272, C273, R274, A275, K276, N277, N278	APOP (frames 1, 3)
		PASSer (frame 2)
		SiteMap (frame 3)
interface	chains A and B: L117, G120, C121, K123, G124, E127, L128, L137, K141, E144, L148	all 9 dimeric frames

a All pockets were identified based on selected frames 1-5 from the monomer simulations and from the frames 1-9 of type 1 dimer.

**Figure 8:**
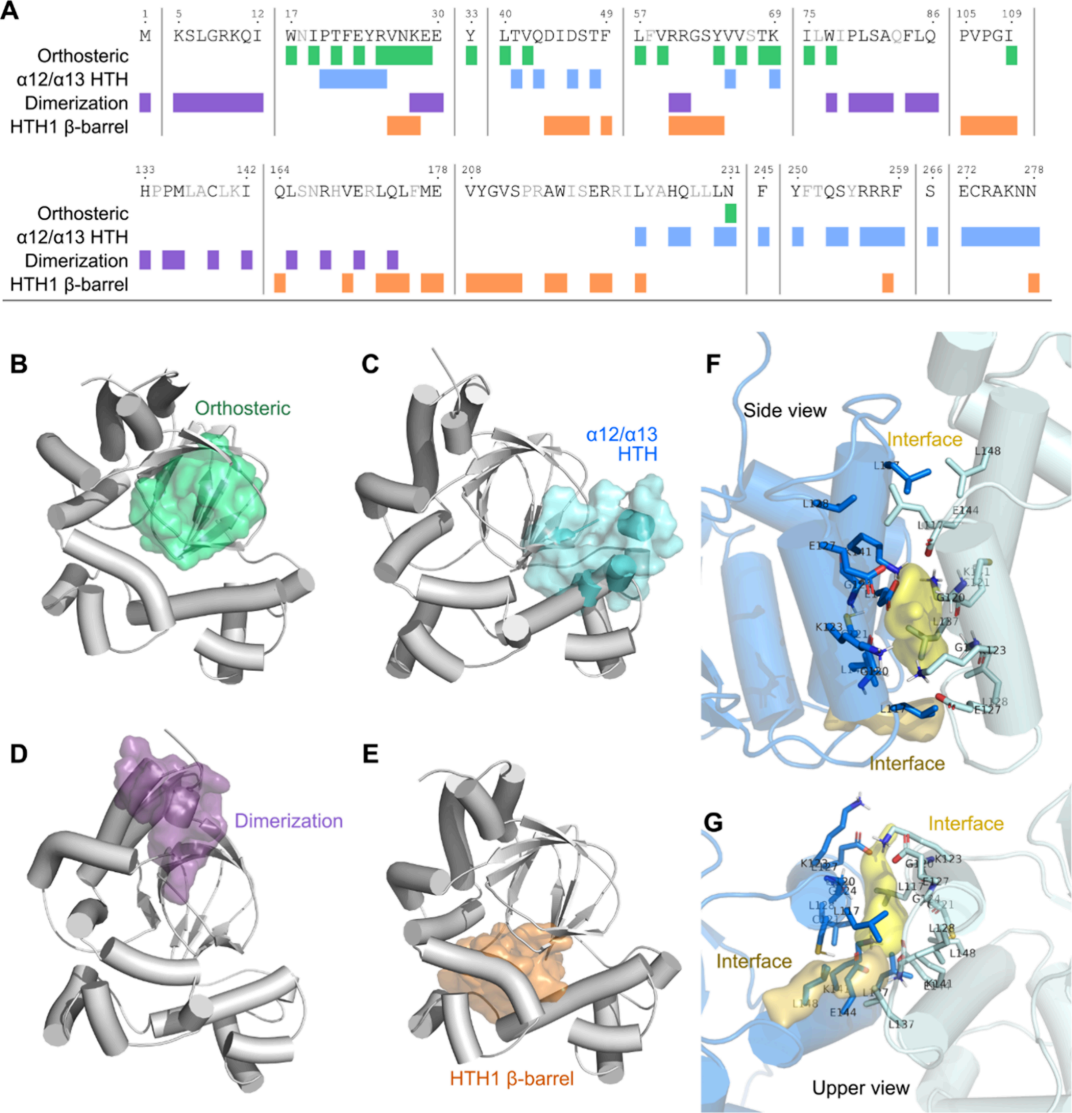
Putative binding sites in the full length ExsA plotted along the primary sequence (A) and pocket surfaces plotted on representative frames for monomeric simulations (B-E). (F,G) Examples of relevant identified interface pockets exclusive for dimer simulations from the “side” (F) and “top” view (G). See Figures S10 and S11 for more details.

As already evident from the pocket definition, the pockets have partial overlap and can connect into larger fused pocket regions. SiteMap identified all established pockets in the monomer simulation frames (Figure S10). The orthosteric pocket (Figure 8B) appears to undergo major reorganization and can no longer be identified as a druggable pocket in frame 3. Major fluctuations are also observed for the α12/α13 pocket (Figure 8C) and the dimeric interface (Figure 8D). In contrast, the HTH1 β-barrel pocket is robustly identified as a druggable site in all analyzed simulation frames (Figure 8E).

Potential protein interfaces that can participate in ligand interactions were additionally predicted with PeSTo. PeSTo recognized three pockets as likely ligand interfaces, with only the dimerization site not being flagged as a potential ligand interface and residues belonging to the HTH1 β-barrel pocket being the most featured. Some limitations observed during the prediction process are discussed in the Supporting Information. The allosteric pocket prediction tools APOP and PASSer also identified similar four pockets predicted by the SiteMap. Lastly, PocketMiner – used to study the potential cryptic pockets – identified the dimerization interface and the HTH1 β-barrel pocket. Interestingly, a large consensus pocket was predicted that the residues were enclosed from all four established pockets.

While the four pockets suggested by literature were successfully identified in simulation results using various tools, major fluctuations and partial fusion of different pockets during the MD, poses a challenge to the robust identification of well-defined individual pockets. An additional fifth pocket was exclusively identified in the dimeric simulations, localized on the interface between subunits. The results underline the major dynamical changes in both monomeric and dimer simulations and suggest that dimerization dramatically impacts pocket integrity of sites defined by the monomer simulation analysis. This is further supported by the distribution of potential cryptic sites across the whole ExsA protein pointing to features associated with a particularly flexible and adaptive system. Surprisingly, SiteMap analysis applied on metadynamics’ relevant structures (i.e., energetic inflection points from the collective variables, Figure S9), was able to retrieve most of the described pockets even if with, on average, lower druggability scores. We hypothesize that longer time scale unbiased simulations or better selected collective variables could shed a better light on the mutation effects over the orthosteric pocket.

We chose to analyze the type 1 dimer with DNA (D_DNA_s1) as our model system (Figure S11), with selected frames displaying small variations on the position of the HTHs motifs and the C-terminal region. Besides the previously four identified pocket types from the monomeric simulations, these dimeric trajectories yielded a new type of pocket (Figure 8 **F,G**, Table 1, and Table S10), namely the interface between subunits, either composed by helices α1/α1’ or α2/α2’.

Functionally, α2 and α3 helices are essential for ExsA dimerization and activity^21^ and identification of binding pockets in this region (*e.g*., “dimerization” and “interface”) complements mutational data.^21^ The fickle presence of the “dimerization” pocket, in the inner part of those helices, and solvent exposure in our predictions would not encourage pursuing it with classical small molecules. On the other hand, the interface pocket between subunits displays the highest scoring among our identified predictions and would be encouraged if a modulator is pursued (Figure S11ED,E and Table S10).

In contrast, chemical intervention at the orthosteric pocket by small molecule ligands could allosterically interfere with ExsA dimerization and subsequently impair transcription activity.^8^ The identified putative orthosteric pocket is orthologous to the known lipid binding pockets in ToxT,^19^ Rns^20^ and VirF.^34^ Indeed, Rns dimer display one protomer in the open form, its NTD and CTD are parted by a groove, with electron density suggesting a bound ligand that could fit decanoic acid.

The orthosteric pocket was recently proven as druggable by small molecules of the C26 series, hydrophobic bromo-thiazole amino derivatives, against the *Salmonella* homologue HilD.^35^ Long simulations with C26-derivatives within the HilD binding pocket showed high ligand flexibility. They report multiple intermittent hydrophobic contacts allowing their compounds to shift their location from the orthosteric site’s β-barrels cavity toward the HTH and back, while retaining a low binding energy. This was further corroborated using STD-NMR, suggesting a large overlap between different potential binding modes.

In ExsA, we observed that the α12/α13 HTH druggable site can extend toward the putative CTD’s orthosteric pocket, consisting of α8, α10, α11, and β2 of the NTD, depending on its conformation. Multiple sequence alignment of ExsA against some AraC family proteins (ToxT, MarA and Rob) supports this extension. This extended pocket coincides, for instance, with the center of the β-barrel and is positioned near the arabinose binding region in the *Ec*AraC. Indeed, *Ec*AraC can be regulated by the N-terminal loop interaction with the HTH, which operates like a lid.^36^ In our current structural understanding of ExsA, where only NTD structures are available, the HTH1-β-barrel interface seemed less relevant since the missing CTD leads to a large solvent-exposed surface, which reduces the druggability potential of these pockets. However, simulation of the full length ExsA model shows a large CTD rearrangement, leading to more frequent detection of this pocket and enhancing its potential as a druggable site.

## 3 Conclusions

Our study builds up on some previous work^24,37^ that used multi-μs all-atom MD simulations of HTH–DNA interactions, aiming to understand their interactions with nonspecific DNA sequences. We herein not only discussed the relevance of the HTH-DNA interactions for PA’s ExsA in the generation of plausible structural models but also used our μs long simulations to identify potential ligand binding pockets. To the best of our knowledge, this is the first attempt at understanding the dynamics between the CTD and NTD domains of ExsA upon DNA binding. Our work recapitulates the orthologous orthosteric pocket present in the β-sheet bundle and known to bind lipids in ToxT and VirF proteins^19,34^ and arabinose in the AraC, but also identified a novel extended region including the helices α12 and α13 to aid future structure-guided drug discovery campaigns. Finally, our study suggests that a single helix-turn-helix motif seems to drive ExsA monomer DNA recognition and stabilize the putative ligand-binding domain.

## 4 Material and Methods

### 4.1 Protein 3D Structure Modeling

ExsA monomer was retrieved from the AlphaFold Protein Structure Database (https://alphafold.ebi.ac.uk/, code: P26993 (EXSA_PSEAE)).^25,38^ All other models were generated using AlphaFold via the AlphaFold Colab resource implemented in Google Cloud. For multimer predictions, we used the AlphaFold variant developed for multimers (AlphaFold.ipynb^25,38^ ), using the default options. All 3D-structural alignments were conducted in PyMOL (v2.5.2–3.1), for ExsA models only the NTD was utilized for alignment due to the variation in CTD positioning.

### 4.2 Molecular Dynamics Simulations

For all structures protonation states of amino acids were optimized with PROPKA (Schrödinger, LLC, New York, NY, 2021), where we selected the most likely ionization state as proposed by the software, and the structures were minimized. The following systems were selected for the simulations: namely monomer (M), monomer with DNA (M_DNA_), Monomer with DNA site1 (M_DNA_s1), Monomer with site 2 DNA (M_DNA_s2), dimer (D_apo_), Dimer type 1 (D_DNA_s1) and type 2 (D_DNA_s2). MD simulations were carried out using Desmond,^39^ with the OPLS4 force-field.^40^ The simulated system encompassed the protein–ligand complexes, a predefined water model (TIP3P^41^) as a solvent and counterions (Na^+^ or Cl^–^ adjusted to neutralize the overall system charge). The system was treated in a cubic box with periodic boundary conditions specifying the shape and the size of the box as 13 Å distance from the box edges to any atom of the protein. We used a time step of 1 fs, the short-range Coulombic interactions were treated using a cutoff value of 9.0 Å using the short-range method, while the smooth Particle Mesh Ewald method handled long-range Coulombic interactions.^42,43^

Initially, the relaxation of the system was performed using Steepest Descent and the limited memory Broyden-Fletcher-Goldfarb-Shanno algorithms in a hybrid manner. Simulations were performed under the NPT ensemble for 5 ns implementing the Berendsen thermostat and barostat methods. A constant temperature of 310 K was kept throughout the simulation using the Nose-Hoover thermostat algorithm^44,45^ and Martyna-Tobias-Klein Barostat algorithm^46,47^ to maintain 1 atm of pressure, respectively. After minimization and relaxation of the system, we continued with the production step of at least 1 μs, depending on the system, with sampling every 1,000 ps. For each system five independent replicas were run totaling around 9.5 μs for M (i.e., monomer without DNA), 12.5 μs for the M_DNA,_ 10 mS for the MDNAs1, MDNAs2, Dapo, DDNAs1 and DDNAs2 systems. The crystal structure of the regulatory domain (NTD domain of ExsA) was simulated for total 5 μs for comparison. Trajectories and raw interaction data are available on Zenodo.

### 4.3 Analysis of MD Simulation Trajectories

#### 4.3.1. Protein–Ligand Interactions and Protein Properties

The Maestro simulation interaction analysis tool (Schrödinger, LLC) was used for the analysis of RMSD, RMSF, and interaction analysis. We used default values for interactions, which are - for H-bonds: cutoff of 2.5 Å for donor and acceptor atoms, donor angle of 120° and acceptor angle of 90°. Hydrophobic interactions: cutoff of 3.6 Å between ligand’s aromatic or aliphatic carbons and a hydrophobic side chain. π-π interactions: two aromatic groups stacked face-to-face or face-to-edge. Water bridge interactions: default cutoff of 2.8 Å for donor and acceptor atoms, donor angle of 110° and acceptor angle of 90°. For angle and distance calculations, the Maestro event analysis tool (Schrödinger, LLC) was used. Distances between specific secondary structure elements were calculated using their centers of mass with the Maestro script *trj_asl_distance.py* (Schrödinger LLC).

#### 4.3.2. Principal Component Analyses (PCA)

PCA of trajectories from the MD simulations was performed to see the overall motion of the protein complexes. All the monomeric trajectories without DNA from the simulations were merged into one trajectory file (total 9.5 μs) using trj_merge.py script and aligned to the initial frame using the trj_align.py script. A python script, trj_no_virt.py provided by Schrödinger was used to convert the.cms and trajectory files to.pdb and.xtc files, which were used as input files for Gromacs (version 2021.4). Extracellular and intracellular loops were excluded and only the transmembrane regions were considered further, using make_ndx script in Gromacs. Gromacs tool gmx covar, was used to calculate and diagonalize the covariance matrix. The eigen vectors produced are analyzed and projected using gmx anaeig script in Gromacs. Mode vectors script from PyMOL v2.5.4 (Schrödinger LCC, New York, NY, USA) was used to visualize the principal components generated by the previous step.

#### 4.3.3. Gibbs Free Energy Landscape Analysis

To investigate energetically favorable conformations during the simulation, Gibbs free energy landscape (FEL) analysis was conducted using the principal components. The FEL was generated in Gromacs using gmx sham module based on projections along the first (PC1) and second (PC2) principal components, respectively. This FEL reflects the relative Gibbs free energy values computed from the MD simulation trajectories based on Boltzmann distributions. FEL was generated for both the monomer and dimer systems to inspect the stable conformational states. The red colored bins indicate the low energy states, and the blue colored bins indicate the high energy states. The lowest energy conformations (or frames) associated with the red bins are extracted using gmx trjconv and then clustered using the gromos clustering method (gmx cluster) with an RMSD cutoff 0.2.

#### 4.3.4. Binding Site Prediction

ExsA binding pockets were predicted using multiple different tools which were interpreted as a consensus using relevant conformations determined according to structural features, such as CTD-NTD opening angle. The monomer frames were extracted based on variations in the angles within the CTD HTH motif region, specifically involving H169, E78, and Q183. These frames were selected to investigate changes in the binding pocket during DNA interaction throughout the simulation. Dimer frames were extracted from the apo simulation, spanning from the reference frame to the point where the protein begins folding into its monomeric form to include the conformational changes.

To determine potential binding sites for drug-like molecules, the Schrödinger SiteMap^48^ tool was used. SiteMap identifies putative binding pockets and evaluates them for favorable interaction hot spots with hydrophobic, hydrogen-bond donating or accepting, and metal-binding ligand functionalities. Sites are evaluated by two metrics: The SiteScore provides the probability of a site being suitable for ligand binding based on volume and enclosure, whereas the DScore (druggability score) emphasizes site druggability by evaluating site hydrophilicity. SiteMap was applied to selected representative frames of the ExsA monomer (5 frames, labeled monomers 1–5) and dimer (9 frames, representing the closest and farthest NTD-CTD distances as well as different conformations of the dimerization interface) with default settings, considering sites with a SiteScore and a DScore >0.8 as potential druggable sites (Tables S2 and S3).

SiteMap was additionally run with modified settings as previously described^49,50^ to screen for potential shallow, hydrophobic sites associated with protein:protein interactions. The modified druggability score DScore+ was calculated as ‘DScore +0.3 phobic’ and pockets with a DScore+ of 1.3 or greater were considered druggable PPI-like pockets (see Table S4).^49,50^ Pocket residues were extracted from SiteMap results with the help of Schrödinger’s Python.

Since SiteMap was originally primarily validated with classical small-molecule druggable sites and corresponding crystallographic evidence, the dynamic nature of ExsA might present a challenge for robust site prediction with SiteMap. The pocket prediction was thus extended by two approaches designed to identify allosteric pockets, the Allosteric Pocket Prediction (APOP) tool and the Protein Allosteric Site Server (PASSer).^51,52^ Allosteric pockets are often associated with major dynamical changes of pocket residues, providing tools designed to detect such pockets with a potential advantage in the ExsA analysis. Both approaches start from site predictions with the pocket prediction tool Fpocket.^53^ APOP employs Gaussian network model (GNM) perturbations to Fpocket predicted sites.^51^ The APOP Web server was used with the default GNM distance cutoff of 10 Å and all chains were considered. Based on visual inspection, a cutoff APOP score of 1.0 was defined to consider allosteric pocket candidates (summarized in Tables S5 and S6). For PASSer, the ensemble of the extreme gradient boosting and the graph convolutional neural network model was used to predict the probability of allosteric sites. Based on visual inspection, pockets with a probability of at least 30% were considered (Tables S7 and S8).^52^

Additionally, two approaches relying on deep learning were applied to the selected ExsA structures. The Protein Structure Transformer (PeSTo) Web server, which employs a parameter-free transformer model, was used to screen for likely ligand- and protein-binding interfaces (summarized in Table S9).^54^ Any residues with scores of 0.5 or greater were classified as part of a predicted binding interface. PocketMiner, which employs a graph neural network was used to identify potential cryptic pockets that are predicted to open in the course of MD simulations.^55^ To define potential cryptic pockets in the PocketMiner results, we employed a minimum score of 0.7 as the cutoff.

Predicted pockets were analyzed visually and classified into four established pockets: (i) the orthosteric pocket, (ii) the dimerization interface, (iii) the HTH1 – β-barrel interface, and (iv) the α12/α13 – HTH pocket. Based on the selected pocket residues (Table 1), all predicted pockets were classified for their overlap with the four different defined pocket regions (percentage of shared residues). The results for all tools are listed in Supplementary data set available in the respective Zenodo repository.

## Supplementary Material



## Data Availability

Prepared structures, MD trajectories, MD simulation configuration and parameter files, as well as raw and processed data for protein–ligand interactions are available in the Zenodo repository (under the doi 10.5281/zenodo.8129269, doi.org/10.5281/zenodo.10518588 and doi.org/10.5281/zenodo.13337568 upon publication). Binding site prediction results are available in the Zenodo repository (under DOI 10.5281/zenodo.15105668). Third-party software employed in the manuscript were as follows. GraphPad PRISM version 10.2 (https://www.graphpad.com/) is distributed under license. Schrödinger Suite 2019–2024.3 (https://www.schrodinger.com) is distributed under license. PyMOL version 2.5.2–3.1.3.1 (https://pymol.org/) is distributed under license.

## Notes

Supplementary figures, data collection, and further information (PDF)

## Abbreviations and Symbols

HTH: helix-turn-helix
MD: molecular dynamics
PDB: Protein Data Bank
T3SS: Type III secretion system
WHO: World Health Organization
WT: wild type
