# Supplementary material for: A Twist of Fate: The Helix–Turn–Helix Motif in Pseudomonas aeruginosa ExsA Can Allosterically Stabilize the Ligand-Binding Domain
Source: J Chem Inf Model. 2025 Nov 11;65(22):12448–63. doi: 10.1021/acs.jcim.5c01120 (PMC12648661; doi:10.1021/acs.jcim.5c01120)
Supplement: Supplementary file 1 [file ci5c01120_si_001.pdf]

SUPPORTING INFORMATION

A twist of fate: the helix-turn-helix motif in *Pseudomonas aeruginosa* ExsA can stabilize the ligand-binding domain

Prasanthi Medarametla[1.3], Jack Calum Greenhalgh[2.3], Ina Pöhner[1], Martin Welch[3], Antti Poso[1.4.5], Thales Kronenberger[1,5*], Taufiq Rahman[2*]

[1] School of Pharmacy. Faculty of Health Sciences. University of Eastern Finland. 70211. Kuopio. Finland.
[2] Department of Pharmacology. University of Cambridge. Tennis Court Road. CB2 1PD. United Kingdom
[3] Department of Biochemistry. Hopkins Building. Tennis Court Road. Downing Site. University of Cambridge. Cambridge. CB2 1QW. United Kingdom
[4] Department of Pharmaceutical and Medicinal Chemistry, Institute of Pharmaceutical Sciences, Eberhard-Karls-Universität, Tübingen, Auf der Morgenstelle 8, 72076 Tuebingen, Germany.
[5] Interfaculty Institute of Microbiology and Infection Medicine (IMIT), University of Tübingen, Tübingen, Germany; Partner-site Tübingen, German Center for Infection Research (DZIF), Elfriede-Aulhorn-Str. 6, 72076 Tübingen, Germany.

*Corresponding authors: T.K.: thales.kronenberger@uni-tuebingen.de. Auf der Morgenstelle 8. DE72076. Tübingen. Germany. T.R.: mtur2@cam.ac.uk. Tennis Court Road. CB2 1PD. United Kingdom

Supporting information

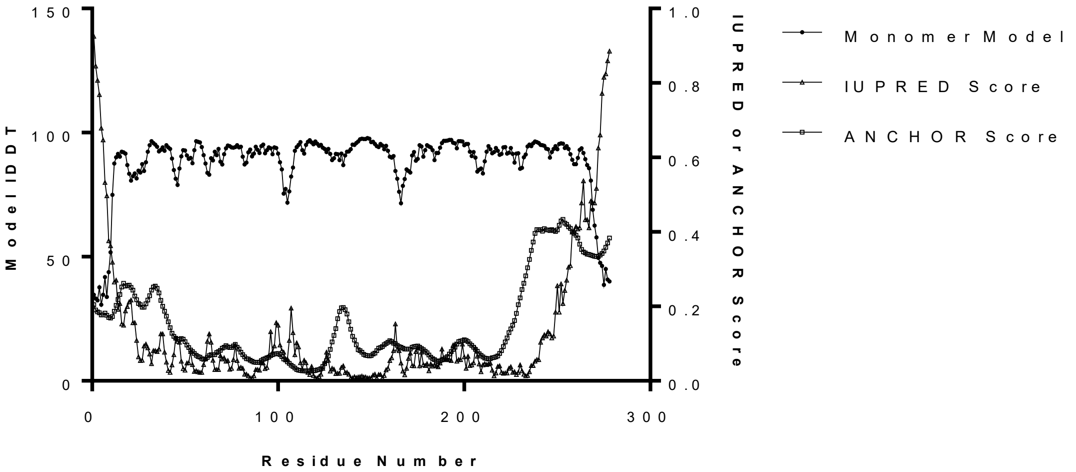

Figure S1. AlphaFold lDDT score plotted for each residue, above the corresponding IUPRED and ANCHOR scores, which are predictive of disorder and stabilization of disordered regions during binding, respectively, generated from the ExsA protein sequence. Each of these areas was also detected as being liable to disorder by IUPred2A though only the extreme terminal sequences met the score threshold of 0.5 to be classed as a disordered region.

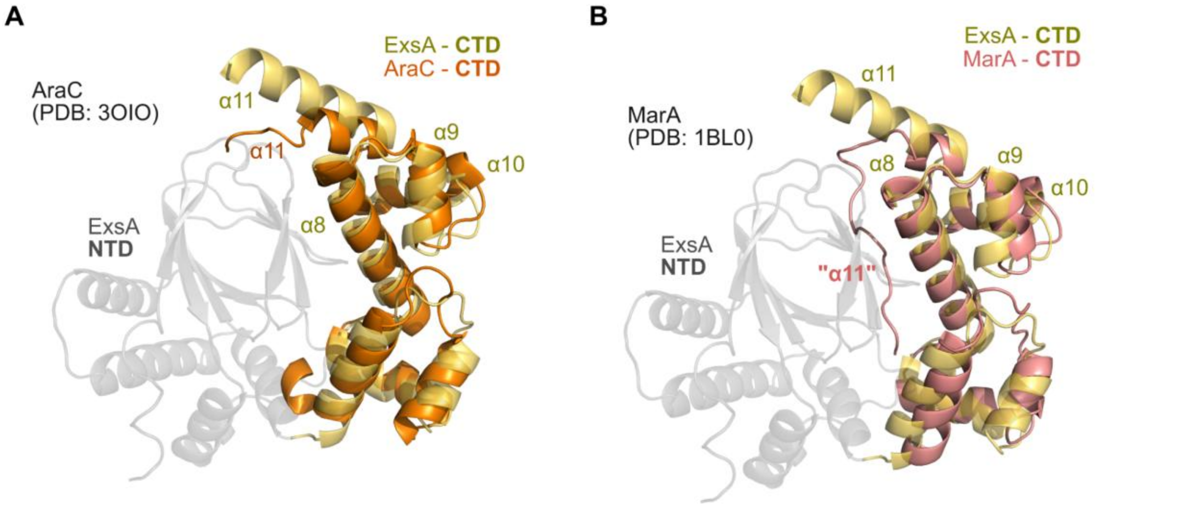

Figure S2. Superimposition of the ExsA model (transparent cartoon) with the CTD relevant crystal structures of AraC from *C. violaceum* (**A**, PDB 3OIO) and the DNA-bound MarA (**B**).

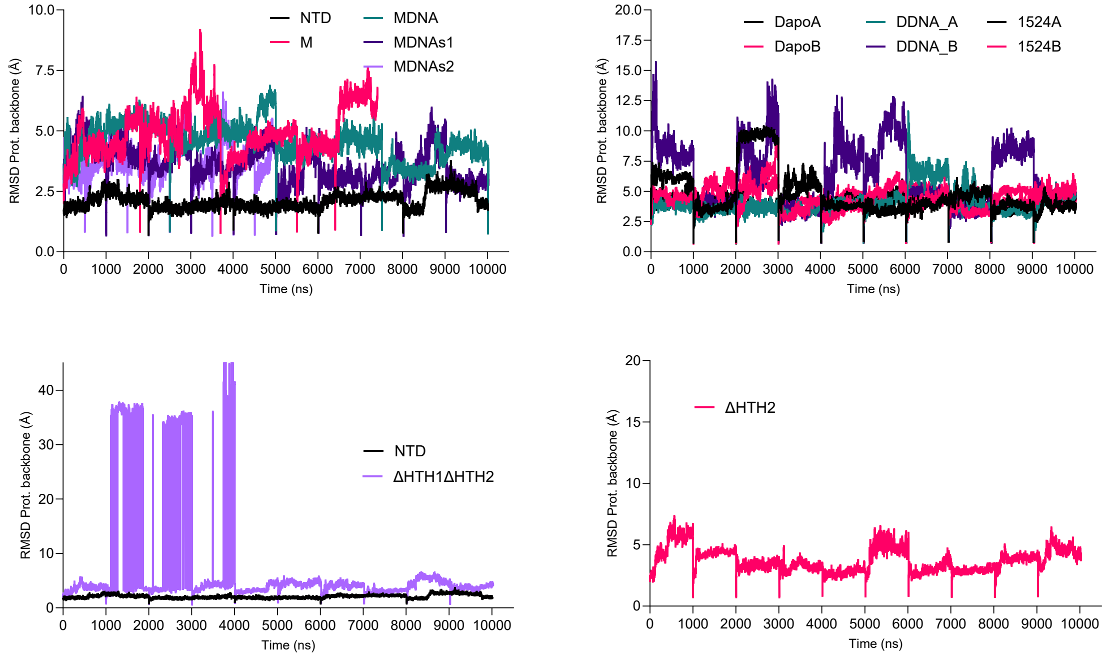

Figure S3. Root mean square deviation (RMSD) of the protein backbone's atoms along the simulation time for each analyzed system, including only NTD to monomer, all monomeric systems with and without DNA and mutants. Bottom panel shows the RMSD of the NTD Vs. mutant HTH region 1 and NTD Vs. mutant HTH region 2.

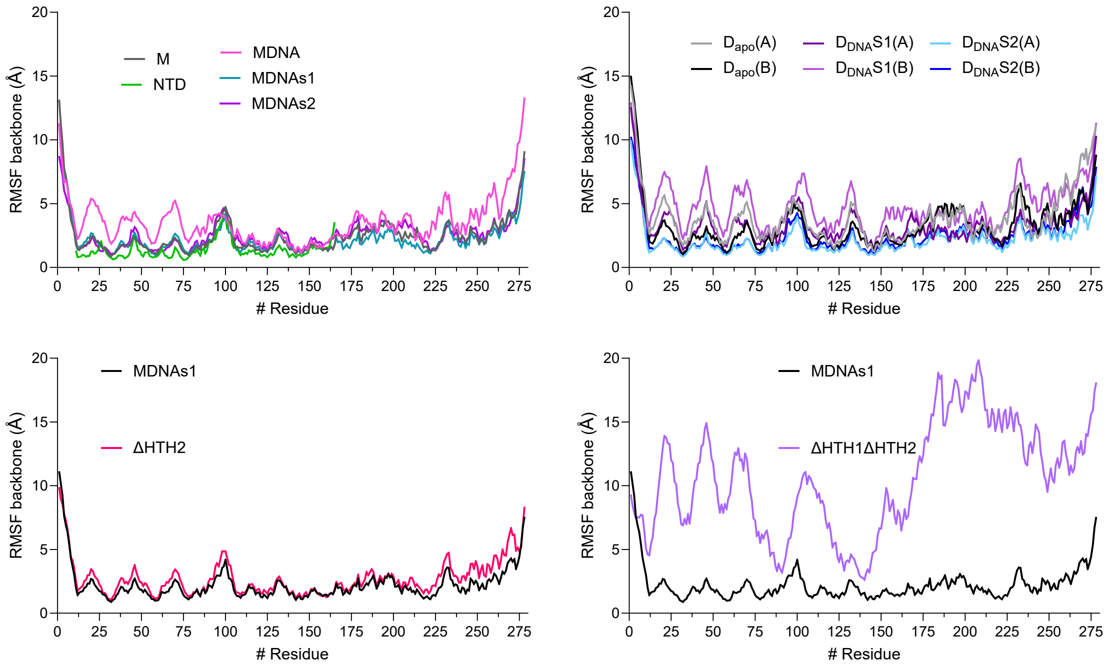

Figure S4. Root mean square fluctuations (RMSF) of the protein backbone's atoms along the simulation time for each analyzed system. Bottom panel shows the RMSFs for simulations with mutants in comparison with the MDNAs1 system.

Table S1. Pairwise distances between the residues located in the NTD during the simulations. Mean and Standard deviation (SD) values are presented. Arg25, Lys28, and Trp77 are in the beta strand subdomain, which is the putative ligand binding domain, in the NTD.

**Dist. Pair.**	**E23-L165**		**I78-C139**		**L80-L128**		**R25-W77**		**K28-W77**	
**System**	Mean	SD	Mean	SD	Mean	SD	Mean	SD	Mean	SD
NTD	30.57	4.85	12.71	4.34	17.07	3.85	12.71	3.23	11.80	1.06
M	29.52	5.30	13.90	4.90	17.27	4.53	12.90	3.73	12.00	0.90
M_{DNA}	28.59	1.71	9.82	0.53	15.74	0.81	12.03	0.83	11.30	1.02
M_{DNA}s1	29.53	2.73	9.95	0.54	16.03	1.20	24.92	1.49	11.50	1.08
M_{DNA}s2	38.93	2.85	9.85	0.82	15.63	1.08	30.58	2.57	11.90	0.92
Dapo(A)	28.6	2.07	10.1	0.73	16.9	1.24	10.5	1.36	11.80	1.08
Dapo(B)	28.1	2.29	10.1	0.70	16.5	1.33	10.9	1.15	11.80	1.06
D_{DNA}s1(A)	28.9	2.34	10	0.70	17.2	1.34	11.1	1.48	12.00	0.90
D_{DNA}s1(B)	27.4	1.99	9.97	0.62	17	1.33	10.7	1.16	11.30	1.02
D_{DNA}s2(A)	28.9	2.14	9.76	0.69	16.5	1.19	11.6	0.85	11.50	1.08
D_{DNA}s2(B)	28.6	2.08	9.85	0.60	16.6	1.08	11.2	0.95	11.90	0.92

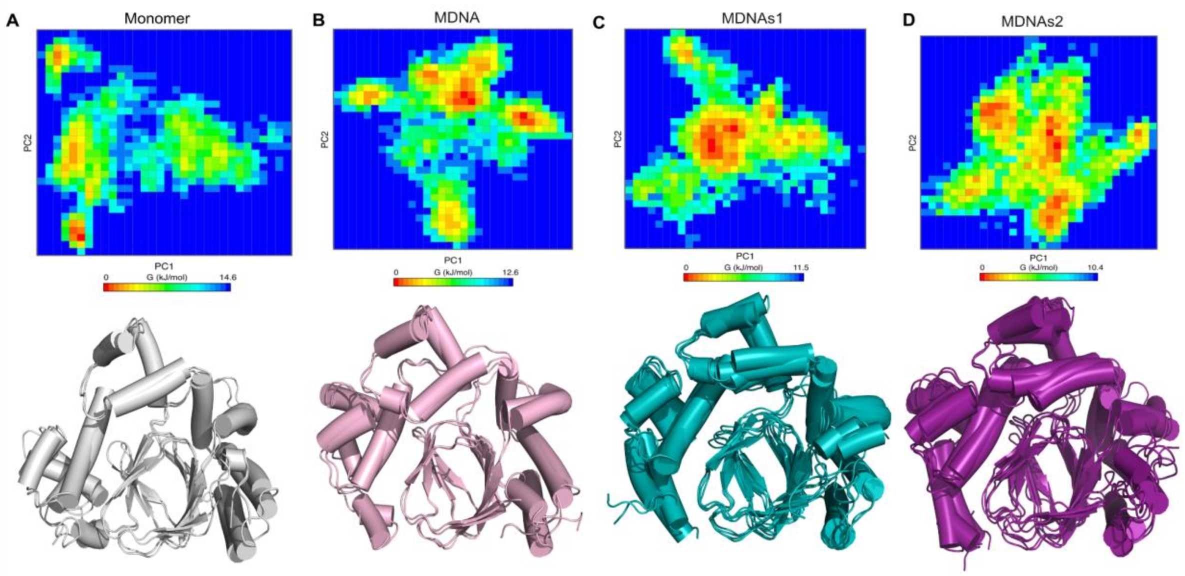

Figure S5. Gibbs free energy landscape analysis. The 2D graphs present the FEL generated from the first and second PCs from PCA analysis. The red indicates low energy and blue indicates high energy. The free energy values are the relative Gibbs free energies derived from the Boltzmann distributions and are not the absolute free energy values. Zero indicates the lowest energy state or conformation. The structures represented along with the 2D graphs are the representative lowest energy states (derived from the red colored bins associated in the graph).

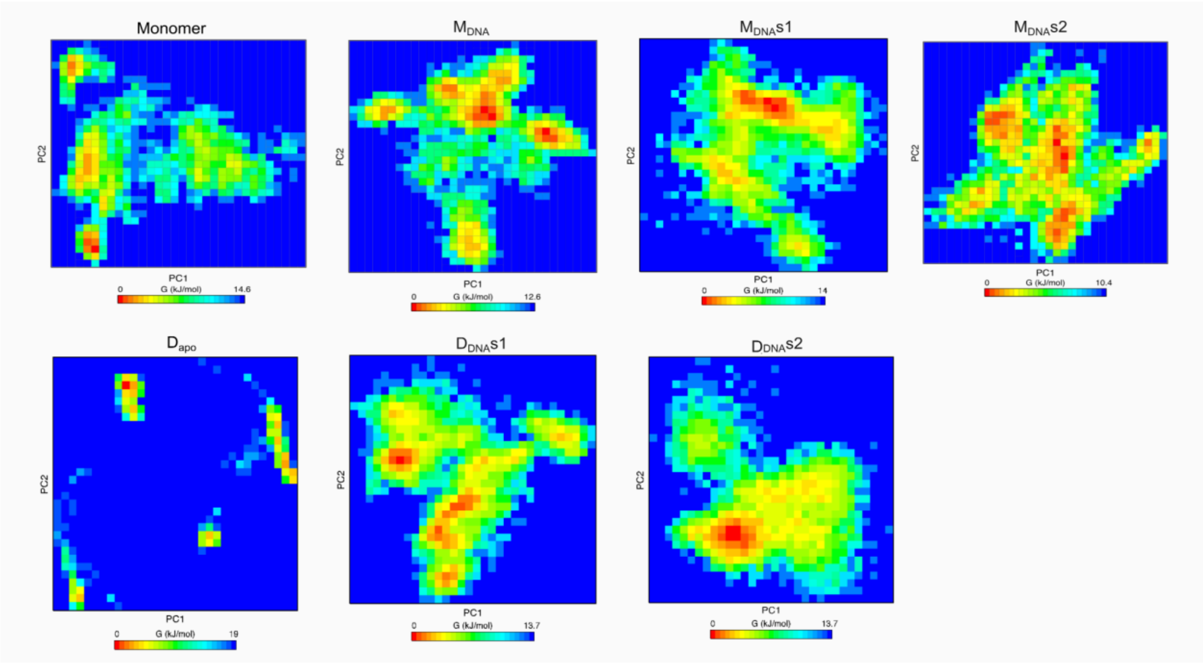

Figure S6. Gibbs free energy landscape analysis. The 2D graphs present the FEL generated from the first and second PCs from PCA analysis. The red indicates low energy and blue indicates high energy. The free energy values are the relative Gibbs free energies derived from the Boltzmann distributions and are not the absolute free energy values. Zero indicates the lowest energy state or conformation.

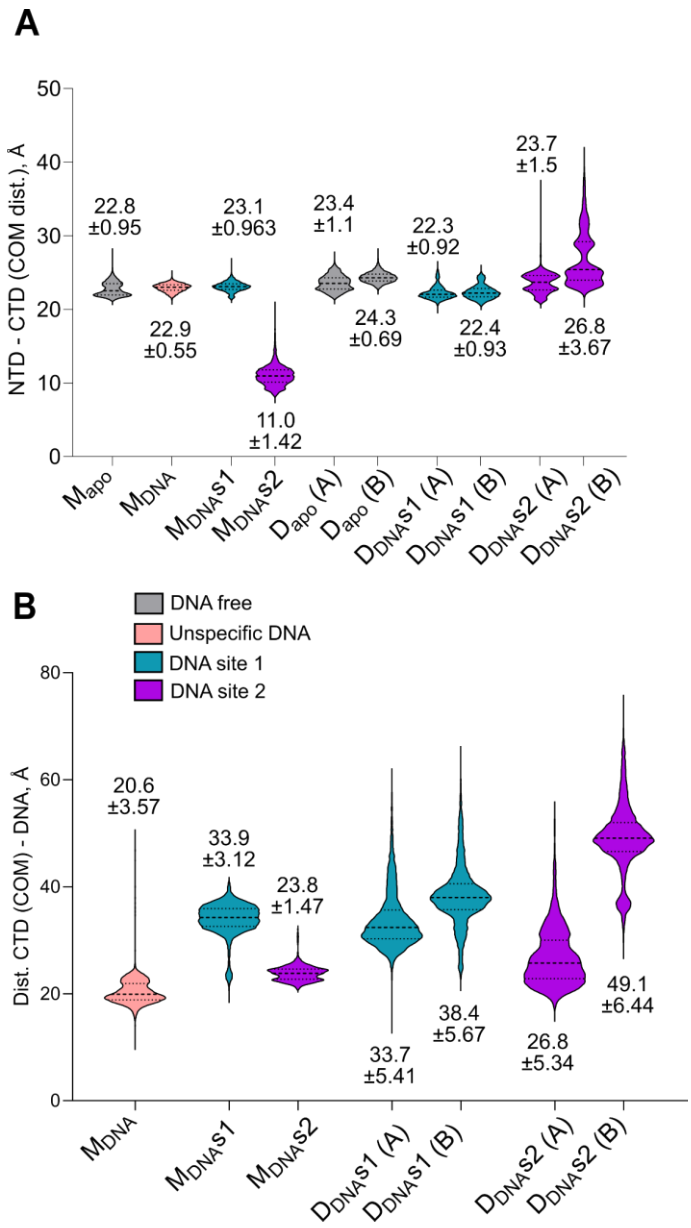

Figure S7. (A) Center of mass (COM) variation from the NTD to the CTD. Mean values are indicated as numbers above the cumulative distribution plots. (B) Center of mass variations are calculated from the CTD region to the DNA. Mean values are represented as numbers below the cumulative distributions.

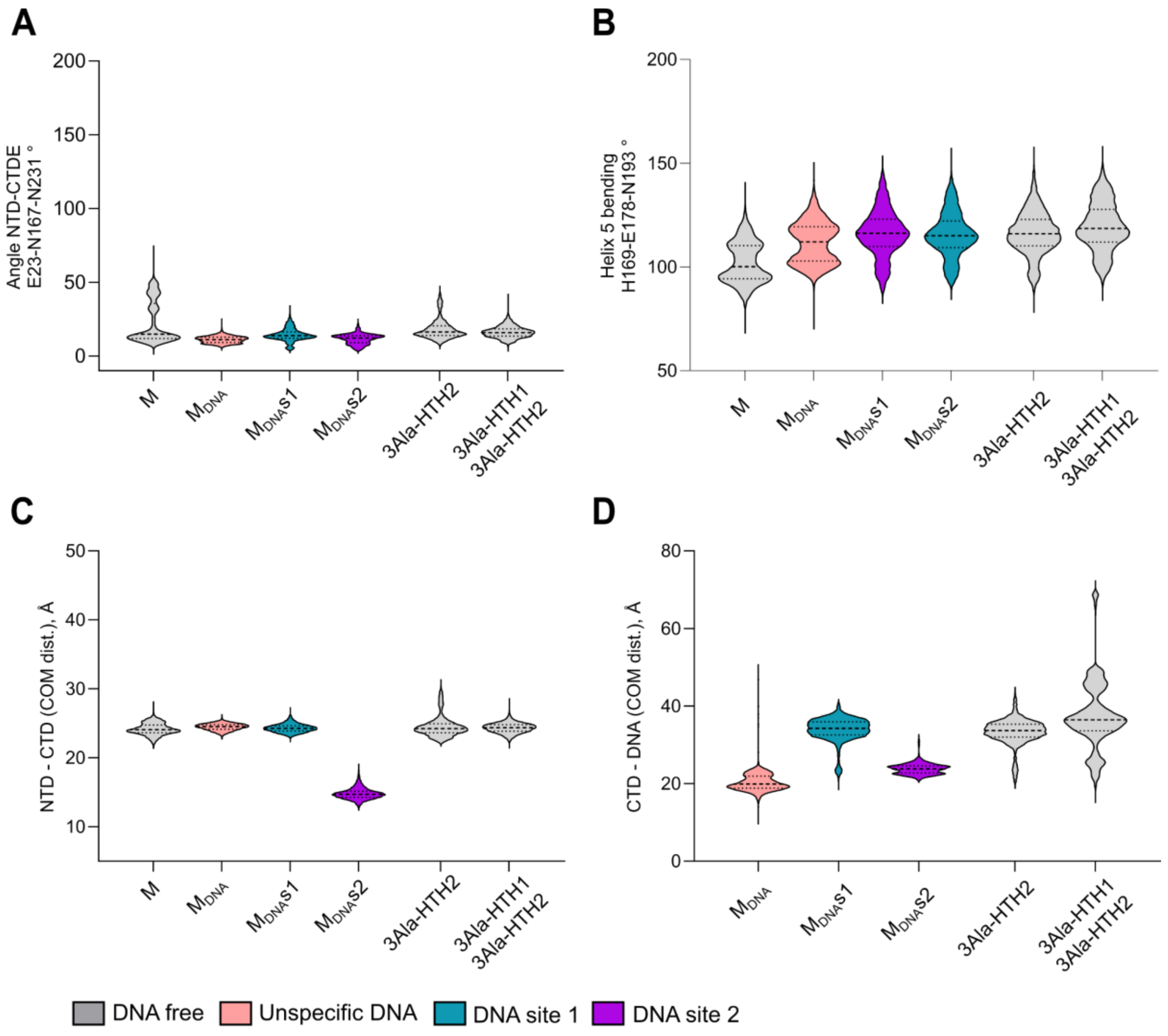

E

		M	M_{DNA}	M_{DNA}S1	M_{DNA}S2	3AlaHTH2	3AlaHTH
**Angle NTD-CTD**	Mean	23	11.1	14.3	11.6	18.2	16.2
**E23-N167-N231**	Std	14.3	2.3	4.4	3.4	6.8	3.9
**H5 bending**	Mean	102	112	116	116	116	119
**H169-E178-N193**	Std	9.9	10.0	10.8	11.3	10.5	11.6
**Distance**	Mean	24.2	24.5	24.3	14.8	24.5	24.3
**NTD-CTD**	Std	0.9	0.5	0.6	0.8	1.5	0.7
**Distance**	Mean		20.6	33.9	23.8	33.3	37.4
**NTD-DNA**	Std		3.6	3.1	1.5	3.4	8.6

Figure S8. Geometric changes in the ExsA monomeric mutants bound to DNA site 1 in comparison to M_{DNA}S1 WT. Metrics such as the changes in the angle between NTD and CTD (A), bending of helix 5 (B), NTD and CTD distances (C) and CTD distance to DNA (D) were evaluated. Refer to main text for their respective discussions and to Table in (E) for their mean and standard deviation values.

Supporting information

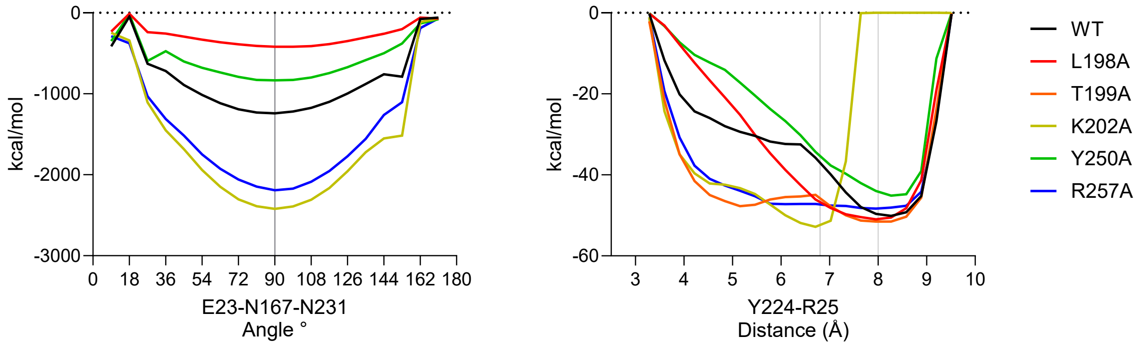

Figure S9. Energy variation (in kcal/mol) distributed along the collective variable (CVs) values' range, for ExsA MDNAS1-WT and respective mutants. Left: angle between E23-N167-N231 and right Y224-R25. CVs were selected based on the angle (A) or distance (B) between the carbon-alpha atoms of the residues. Metadynamics simulations were calculated using Desmond, same conditions as classical MD simulations (see methods), using the module implemented in Maestro 2024v3.

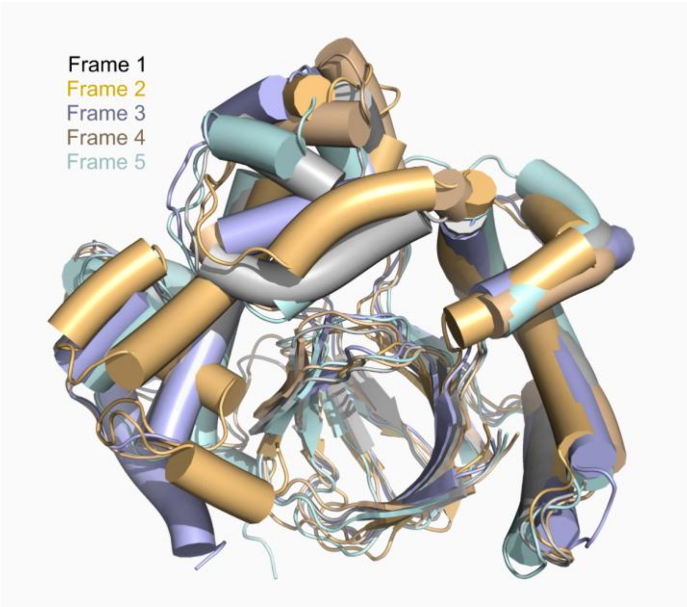

Figure S10. ExsA MD analysis snapshots or frames utilized to analyze the possible druggable binding pockets for monomers.

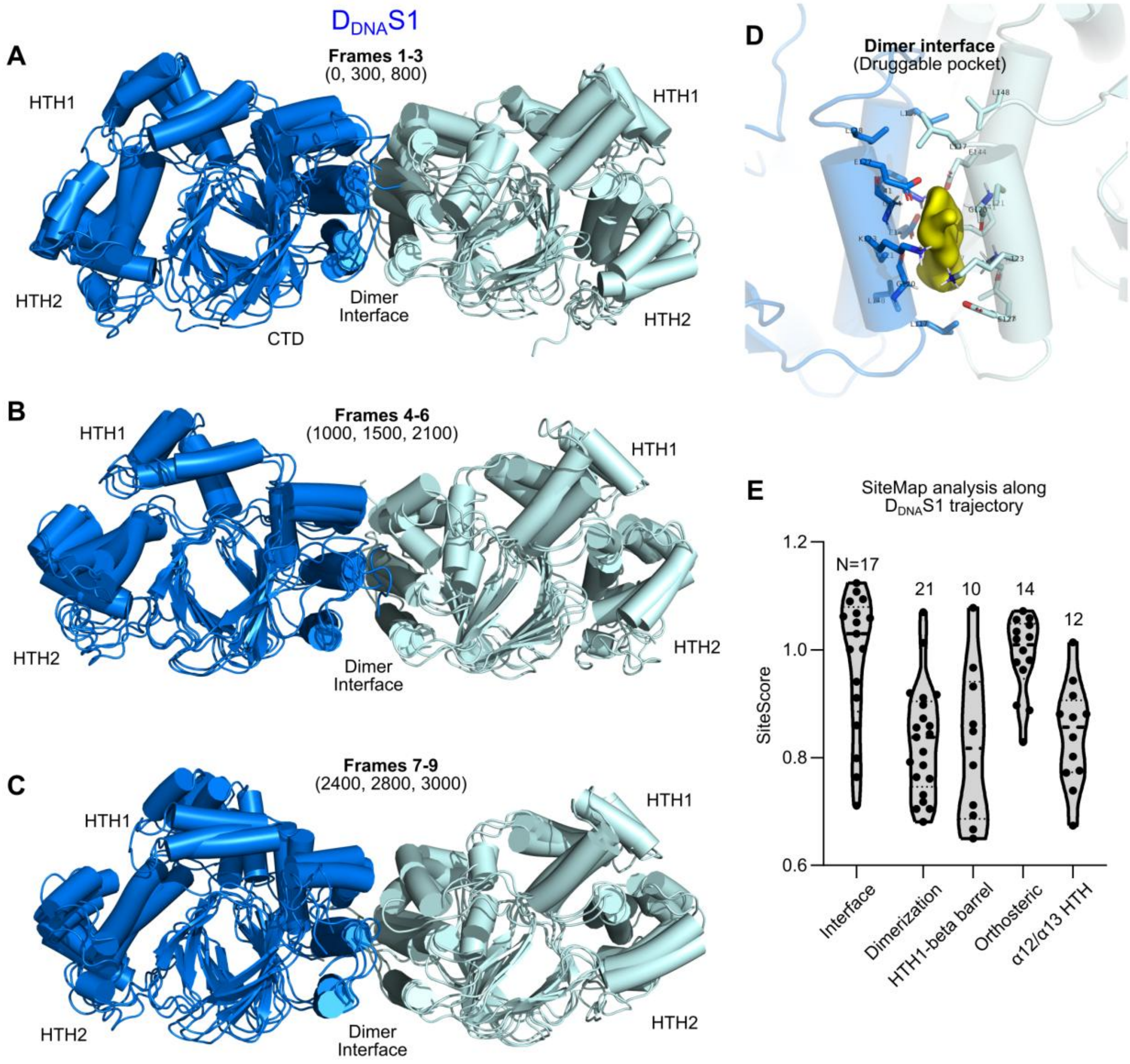

Figure S11. ExsA MD analysis snapshots or frames utilized to analyze the possible druggable binding pockets for dimers divided into three groups: frames 1-3 (**A**), frames 4-6 (**B**), frames 7-9 (**C**). (**D,E**) newly identified dimerization interface druggable pocket, visualized in the 3D structure inset (**D**) and **E**) Distribution of the SiteScores for each relevant identified pocket along the type 1 dimer simulations, N represents the number of identified pockets.

Supporting information – extended results for Potential druggable binding pockets and hotspots on ExsA. SiteMap was also used to determine potential small molecule-druggable PPI sites by using a modified set of parameters. Conversely, while the orthosteric (partially fused with the dimeric interface) and the HTH1 beta-barrel pockets are identified as druggable PPI sites in some frames, no pockets of sufficient druggability are determined in the first and last analyzed frame, again pointing to the high fluctuations the system undergoes. In line with the classical SiteMap prediction, the analysis of frames from dimer simulations with modified parameters did not determine overlapping pockets in line with the literature-based definitions. PeSTo was also used to predict potential protein interfaces. In the first analyzed frame, residues from all pockets are predicted potential protein interfaces, after which minor overlap with the α12/α13 HTH pocket and the dimeric interface are predicted as protein interfaces. No probable protein interfaces were detected in the dimer frames.

Table S2. Predicted pockets using Schrödinger SiteMap[1,2] with default parameters for selected representative frames from monomer simulations. All druggable pockets (DScore of at least 0.8) are reported as ranked by SiteMap (based on the SiteScore). Phobic: Hydrophobicity score of the binding site.[2]

Analyzed frame	Pocket rank	DScore	Volume [Å^3]	Phobic	Pocket residues
1	1	1.047	229.81	0.463	F22, T41, V42, Q43, D46, S47, T48, V65, V66, K69, L223, H226, R258, F259, C273, R274, A275, K276, N277, N278
1	2	0.985	278.86	0.260	D44, I45, D46, S47, F49, F58, R60, Y64, P105, V106, P107, G108, I109, V161, L173, Q174, M177, S207, Y209, G210, V211, S212, A215, W216, E219, R220
1	3	0.829	128.28	0.578	W17, I19, R25, V26, N27, K28, E29, Y33, L40, V42, V59, Y64, V66
2	1	1.013	412.63	0.611	D44, I45, D46, S47, F49, R60, G62, S63, Y64, P105, V106, P107, G108, I109, I110, L157, L173, Q174, M177, Y209, G210, V211, S212, A215, W216, E219, R220, R258, N278
2	2	1.039	219.18	1.643	W17, I19, R25, V26, N27, K28, E29, Y33, L40, V42, L57, V59, Y64, V66, T68, I75
2	3	0.872	181.10	0.306	K10, Q11, E29, E30, R60, R61, P79, L80, S81, A82, F84, L85, Q86, I142, L165, R168, E171, Q174
3	1	0.812	224.66	0.610	P20, T21, F22, E23, Y24, V65, L223, H226, Q227, L230, N231, R258, F259, S266, E272, C273, N277
3	2	0.899	219.18	0.923	Y24, V26, K28, I45, R61, G62, S63, Q174, L175, E178, V208, Y209, W216, R220, Y224

4	1	1.043	1208.73	0.595	M1, Q2, Y24, R25, V26, N27, K28, E29, E30, D44, I45, F58, V59, R60, R61, G62, S63, Y64, W77, L80, S81, A82, Q83, L85, Q86, V106, P107, G108, I109, V161, L162, Q164, L165, R168, V170, E171, Q174, M177, E178, V208, Y209, G210, V211, W216, S218, E219, R220, I222, L223, A243, F245, S254, R257, R258, F259, G260, E272, N277, N278
5	1	1.024	266.51	0.476	D44, I45, D46, R60, R61, G62, S63, Y64, P105, V106, P107, G108, I109, V161, Q164, Q174, M177, V208, Y209, W216, E219, R220, L223, R258
5	2	0.940	162.58	0.810	L95, E98, V99, E100, R101, C102, E104, V106, P107, P156, L157, M159, S160, R163
5	5	0.802	110.79	1.021	W17, R25, V26, N27, K28, E29, Y33, V42, L57, V59, Y64, V66

Table S3. Predicted pockets using Schrödinger SiteMap[1,2] with default parameters for selected representative frames from dimer simulations. All druggable pockets (DScore of at least 0.8) are reported as ranked by SiteMap (based on the SiteScore). Phobic: Hydrophobicity score of the binding site.[2]

Analyzed frame	Pocket rank	DScore	Volume [Å^3]	Phobic	Pocket residues
1	1	0.942	209.23	0.671	Chain A: L36, G54, L56, A114, P116, L118, A119, G120, V122, K123, G124, K126, E127<br>Chain B: L36, G54, G120, V122, K123, G124
1	2	0.801	160.87	0.451	Chain A: R214, A215, W216, S218, E219, I222, Y250, S254, R257, R258, K276, N277, N278
2	1	1.160	278.17	1.584	Chain A: L117, G120, K123, G124, E127, L128, L137, K141, E144, L148<br>Chain B: P116, L117, A119, G120, C121, K123, G124, E127, L128, L137, K141, E144, L148
2	2	1.025	240.44	0.579	Chain A: L182, R221, M241, E242, G244, F245, S246<br>Chain B: W185, K186, L187, S188, L198, K202, E203, F205, G206, P213, I217, R220, R221, Y224
2	3	0.821	174.24	0.449	Chain A: V89, Q90, G93, L96, V99, E100, R163, S166, N167, R172, L175, F176, K179, H180, E193, F194, M196
2	4	0.906	239.07	0.881	Chain A: Q2, R9, R91, F92, P135, M136, C139, L140, E143<br>Chain B: R91, F92, C139, L140, E143, M147
3	1	1.020	1526.35	0.674	Chain A: R9, K10, Q11, E29, E30, F58, R60, W77, P79, L80, S81, A82, F84, L85, G87, Q90, R91, F92, A94, L140, I142, E143, L146, M147, F149, A150, L158, V161, L162, Q164, L165, R168, H180, E184, W185, K186, R192, E193<br>Chain B: R9, K10, Q11, E29, E30, I78, P79, L80, S81, Q83, F84, G87, F88, R91, F92, L95, M136, L140, I142, E143, M147, H169, R172, Y181, E184, W185, K186, L187, S188, F190, F194, G195, M196, G197, L198, T199, T200, K202, E203, G206, S207, S212, P213, R214, I217, S218, R221, I222, A243, G244
3	2	1.081	296.35	1.241	Chain A: L117, G120, C121, K123, G124, E127, L128, L137, C139, K141, E144, L148, N183, D189<br>Chain B: L117, G120, C121, G124, L125, E127, L128, L137, C139, K141, E144, N183

Table S4. Predicted pockets using Schrödinger SiteMap with modified parameters to predict protein:protein interaction sites and potential cryptic pockets[1,2,3] in selected representative frames from monomer and dimer simulations. All druggable pockets (DScore+ of at least 1.3) are reported as ranked by SiteMap (based on the SiteScore). Phobic: Hydrophobicity score of the binding site.[2]

**Analyzed frame**	**Pocket rank**	**DScore+**	**Volume [Å^3]**	**Phobic**	**Pocket residues**
Monomer					
2	3	1.524	238.17	1.480	W17, I19, R25, V26, N27, K28, E29, Y33, L40, V42, L57, V59, Y64, V66, T68, I75
3	2	1.370	209.62	1.281	Y24, V26, N27, K28, R60, R61, G62, S63, G108, E171, Q174, L175, M177, E178, F205, V208, Y209, W216, R220, Y224
4	1	1.303	1304.00	0.828	M1, Q2, L7, I19, Y24, R25, V26, N27, K28, E29, E30, Y33, D44, I45, V59, R60, R61, G62, S63, Y64, V66, W77, P79, L80, S81, A82, Q83, L85, Q86, V106, P107, G108, I109, V161, L162, Q164, L165, R168, V170, E171, Q174, M177, E178, V208, Y209, G210, V211, W216, S218, E219, R220, I222, L223, A243, F245, S254, R257, R258, F259, G260, E272, N277, N278

Table S5. Predicted allosteric pockets for monomer frames with APOP.[4] An APOP score cutoff of 1.0 was defined for considered pockets. The pocket rank is based on the original pocket score from fpocket.[5] The reported Volume is based on the occupied volume by all alpha spheres in the pocket using a Monte Carlo (MC) algorithm in fpocket. The Hydrophobicity Score is derived from a per-residue hydrophobicity scale by fpocket.[5]

Analyzed frame	Pocket rank	APOP score	Volume (MC)	Hydrophobicity Score	Pocket residues
1	1	1.749	636.46	17.76	F22, T41, Q43, D44, I45, D46, S47, T48, Y64, V65, K69, P105, V106, G108, L173, Q174, M177, L223, H226, R258, F259, C273, R274, A275, K276, N277, N278
1	3	1.263	520.92	1.75	K10, Q11, K28, E29, E30, R61, P79, L80, S81, A82, E171, L175
1	10	1.171	440.72	31.53	V208, Y209, G210, V211, W216, R220
1	4	1.073	175.94	98.00	F58, L80, L85, F88, L146, L162, L165
1	2	1.070	440.74	48.23	W17, I19, R25, V26, N27, K28, E29, Y33, V42, V59, Y64, V66, W77
2	3	2.242	400.64	65.93	W17, I19, R25, V26, N27, Y33, L40, V42, L57, V59, Y64, V66, T68, I75, I109
2	1	1.959	991.23	21.71	S6, R9, K10, Q11, T13, E29, E30, R60, W77, P79, L80, S81, A82, F84, L85, Q86, C139, I142, L165, R168, E171
2	15	1.647	978.83	21.09	D44, I45, D46, S47, R60, Y64, P105, V106, G108, V170, L173, Q174, M177, Y209, G210, V211, S212, A215, W216, E219, R220, R258, N278
3	3	2.794	644.31	15.15	P20, T21, F22, H226, L230, C261, S266, G269, D271, E272, C273, A275, K276
3	1	2.415	643.31	30.12	T21, F22, E23, Y24, R25, Q43, V65, L223, H226, Q227, L230, N231, R258, F259, C273, R274
3	2	1.140	617.00	21.85	V26, N27, K28, R61, G62, S63, Q174, L175, E178, V208, Y209, Y216, R220
4	19	3.634	2282.62	31.28	V26, N27, K28, E30, D44, I45, V59, R60, R61, G62, S63, Y64, L80, A82, L85, P105, V106, G108, I109, V161, L162, Q164, L165, V170, E171, L173, Q174, M177, V208, Y209, G210, V211, W216, E219, R220, L223, H226, R258, F259, G260, E272, N277, N278
5	2	2.470	890.94	19.31	D44, D46, S47, F49, R60, R61, G62, S63, Y64, P105, V106, P107, G108, I109, Q164, V170, Q174, M177, V208, Y209, G210, W216, E219, R220, L223, R258
5	1	1.174	891.99	21.87	M1, K5, L7, G8, R9, K10, I12, T13, E29, W77, P79, H133, P135, M136, C139
5	4	1.048	572.43	9.27	K28, E29, E30, R60, R61, L80, S81, A82, Q83, Q86, L165, R168, E171, Q174, L175
5	8	1.040	503.27	20.33	L95, L96, E98, V99, E100, R101, G155, P156, M159, S160, R163

Table S6. Predicted allosteric pockets for dimer frames with APOP.[4] An APOP score cutoff of 1.0 was defined for considered pockets. The pocket rank is based on the original pocket score from fpocket.[5] The reported Volume is based on the occupied volume by all alpha spheres in the pocket using a Monte Carlo algorithm in fpocket. The Hydrophobicity Score is derived from a per-residue hydrophobicity scale by fpocket.[5]

Analyzed frame	Pocket rank	APOP score	Volume (MC)	Hydrophobicity Score	Pocket residues
1	23	1.857	362.97	13.67	Chain A: Q90, G197, L198, T199, T200, E203<br>Chain B: Q90, T199, T200, E203
1	2	1.265	393.07	3.30	Chain A: P116, A119, G120, K123, G124, E127, K141<br>Chain B: P116, L117, A119, G120, C121, K123, G124, E127, E144
1	4	1.233	421.39	33.50	Chain B: L85, Q86, V89, Q90, L162, R163, L165, S166, T200, Q203, L204, S207
1	16	1.088	382.95	15.54	Chain A: Q11, Q83, F84, Q86, G87, F88, Q90, E143, G195, M196, G197, T200<br>Chain B: R91
1	32	1.077	283.65	51.18	Chain A: F112, A113, T115, L117, L118, L148, F149, F151, S152<br>Chain B: E132, P134
1	1	1.047	400.64	65.93	Chain A: S212, R214, A21, S218, E219, I222, Y250, Q253, S254, R257, R258, K276, N278
2	6	2.226	690.10	19.81	Chain A: L95, E98, T115, P116, K117, M147, L148, F151, S152, P153<br>Chain B: H131, E132, H133, P134, P135, M136
2	4	1.865	300.11	33.69	Chain A: R221, M241, G244, F245, S246<br>Chain B: E184, W185, K186, L187, S188, I217, R220, R221, Y224
2	1	1.792	751.56	23.58	Chain A: L117, G120, K123, G124, E127, L128, L137, K141, E144, L148<br>Chain B: P116, L117, G120, C121, K123, G124, E127, L128, L137, K141, E144, L148
2	9	1.679	249.58	42.83	Chain A: L182, R221, Y224, M241, E242<br>Chain B: L187, L198, F201, K202, F205, G206, P213
2	41	1.154	407.08	37.29	Chain A: R9, R91, F92, M136, C139, L140, E143<br>Chain B: R91, F92, L140, E143
2	40	1.005	347.51	19.50	Chain A: E184, A215, S218, E219, I222, A243, G244, F245, S254, R258
3	7	3.782	863.54	10.90	Chain A: F176, H180, N183, E184, W185, D189, R192, E193<br>Chain B: L187, L198, T199, T200, K202, E203, G206, S207, V211, S212, P213, R214
3	32	2.115	621.76	15.33	Chain A: Q90, R91, A94, E143, R192, E193<br>Chain B: F84, P135, M136, C139, L140, E143, E203

3	1	1.247	151.31	87.80	Chain A: V32, F58, L80, L85, L146, F149, A150, L158, V161, L162
3	5	1.156	574.38	33.15	Chain A: W185, K186, L187, R214, A215, I217, S218, R221, G244, F245, S246, S247, Y250

Table S7. Predicted allosteric pockets for monomer frames with PASSer.[6] A PASSer allosteric pocket probability of 30% was chosen for considering pockets. The pocket rank is based on the original pocket score from fpocket.[5] The reported Volume is based on the occupied volume by all alpha spheres in the pocket using a Monte Carlo (MC) algorithm in fpocket. The Hydrophobicity Score is derived from a per-residue hydrophobicity scale by fpocket.[5]

Analyzed frame	Pocket rank	PASSer probability	Volume (MC)	Hydrophobicity Score	Pocket residues
1	1	41.37	639.56	17.76	F22, T41, Q43, D46, T48, V65, K69, L223, H226, R258, F259, C273, R274, A275, K276, N277, N278
2	1	56.55	1005.95	21.71	S6, R9, K10, Q11, T13, E29, E30, R60, W77, P79, L80, S81, A82, F84, L85, Q86, C139, I142, L165, R168, E171
2	15	37.92	988.75	21.09	D44, I45, D46, S47, R60, Y64, P105, V106, G108, V170, L173, Q174, M177, Y209, G210, V211, S212, A215, W216, E219, R220, R258, N278
2	6	34.28	759.21	12.89	E219, F245, Y250, Q253, S254, R256, R257, R258, A275
2	3	33.11	406.42	65.93	W17, I19, R25, V26, N27, Y33, L40, V42, L57, V59, Y64, V66, T68, I75, I109
3	1	58.84	654.53	30.12	T21, F22, E23, Y24, R25, Q43, V65, L223, H226, Q227, L230, N231, R258, F259, C273, R274
3	2	47.85	604.75	21.85	V26, N27, K28, R61, G62, S63, Q174, L175, E178, V208, Y209, W216, R220
3	3	36.11	642.36	15.15	P20, T21, F22, H226, L230, C261, S266, G269, D271, E272, C273, A275, K276
4	19	65.82	2278.09	31.28	V26, N27, K28, E30, D44, I45, V59, R60, R61, G62, S63, Y64, L80, A82, L85, P105, V106, G108, I109, V161, L162, Q164, L165, V170, E171, L173, Q174, M177, V208, Y209, G210, V211, W216, E219, R220, L223, H226, R258, F259, G260, E272, N277, N278
5	1	84.33	927.28	21.87	M1, K5, L7, G8, R9, K10, I12, T13, E29, Y77, P79, H133, P135, M136, C139
5	2	38.05	880.04	19.31	D44, D46, S47, F49, R60, R61, G62, S63, Y64, P105, V106, P107, G108, I109, Q164, V170, Q174, M177, V208, Y209, G210, W216, E219, R220, L223, R258

Table S8. Predicted allosteric pockets for dimer frames with PASSer.[6] PASSer does not support input with multiple chains and chains A and B of the dimer frames were split and analyzed separately. A PASSer allosteric pocket probability of 30% was chosen for considering pockets. The pocket rank is based on the original pocket score from fpocket.[5] The reported Volume is based on the occupied volume by all alpha spheres in the pocket using a Monte Carlo (MC) algorithm in fpocket. The Hydrophobicity Score is derived from a per-residue hydrophobicity scale by fpocket.[5]

Analyzed frame	Pocket rank	PASSer probability	Volume (MC)	Hydrophobicity Score	Pocket residues
1-A	1	35.38	518.23	4.15	S212, R214, A215, S218, E219, I222, Y250, Q253, S254, R257, R258, K276, N278
1-B	1	39.21	427.62	33.50	L85, Q86, V89, Q90, L162, R163, L165, S166, T200, E203, L204, S207
2-B	1	53.79	778.26	34.46	E55, A111, F112, A113, T115, P116, L117, L118, L148, F151, S152, P153, Q154
3-A	4	40.07	378.77	47.40	E30, F58, R60, L80, L85, V106, V161, Q164, L165, R168
3-A	1	36.45	586.64	33.15	W185, K186, L187, R214, P215, I217, S218, R221, G244, F245, S246, S247, Y250
3-A	2	32.69	149.00	87.80	V32, F58, L80, L85, L146, F149, A150, L158, V161, L162
3-A	3	31.22	473.42	36.15	C15, W17, E23, R25, V26, N27, K28, E29, Y33, Y64, V65, V66, W77

Table S9. Predicted interface residues by PeSTo.[7] PeSTo returns modified PDB-files with interface residues marked in the B-factor column by a value of 0.5 or greater. No protein interface residues were predicted for monomer frame 5 or any of the dimer frames.

Analyzed frame	Type of interface	Interface residues
Monomer		
1	Ligand	K69, H169, K179, N183, L187, G195, M196, S212, P213, R214, A215, R274, N277, N278
1	Protein	Q2, K10, C15, H16, W17, I19, Y24, R25, V26, N27, K28, E29, Y33, V66, I75, W77, R91, F92, A94, L117, M136, L140, E143, E144, F151
2	Ligand	N183, W185, T199
2	Protein	E98, W185, R192, V237, D238, S246, S247, Y250
3	Ligand	R25, R168, H169, T199, S212, R214, R258
3	Protein	M1, Q2, R91, F92, A94, L95, E98, L117, G120, H131, H133, M136, L137, L140, K141, E143, E144, M147, L148, F151
4	Ligand	W17, R25, E30, D44, I45, R60, R61, G62, S63, K179, H180, Y181, Y209, G210, V211, W216, Y224, Q227, N231
4	Protein	R9, R91, V130, H131, P134, P135, M136, L137, L140, N278
5	Ligand	K186, L187, S188, L198, T199, S212, P213, R214, A215
Dimer		
1	Ligand	Chain A: K186, L187, G197, L198<br>Chain B: C15, R25, R214
2	Ligand	Chain A: T13, C15, W17, I19, R25, V26, N27, K28, E29, E30, Y33, V59, R61, Y64, V66, W77, P79, S81, A82, E184, W185, K186, L187, S188, D189, R192, S212, P213, R214, A215, R221, G244, F245, S246<br>Chain B: W185, K186, L187, S188, D189, R192, G195, Y224
3	Ligand	Chain A: R25, V26, N27<br>Chain B: R9, Q11, F84, T115, G197, T199, T200

Table S10. Predicted pockets using Schrödinger SiteMap with modified parameters to predict protein : protein interaction sites and potential cryptic pockets[1,2,3] in selected representative frames from monomer and dimer simulations. All druggable pockets (DScore of at least 0.6) are reported as ranked by SiteMap (based on the SiteScore).

Site_Rnk	Pocket type	Trj frame	Score	Dscore	Volume (Å3)	Residues
site10_1	Interface	0	1.108	1.134	172.529	Chain A: 117,120,121,123,124,127,141,144,148; Chain B: 117,120,121,123,124,127,128,137,141,144
site19_2	Orthosteric	0	1.056	1.087	211.288	Chain B: 43,44,45,46,47,49,60,61,62,63,64,65,105,106,107,108,109,110,111,154,174,178,208,209,210,211,216,219,220,223
site51_3	Dimerization	0	0.873	0.862	158.809	Chain A: 8,9,10,11,12,13,14,128,129,133,135,138,139,142
site58_4	Dimerization	0	0.859	0.850	254.849	Chain A: 91,92; Chain B: 9,10,11,12,13,79,80,81,84,87,88,91,128,129,133,135,136,138,139,143
site73_5	HTHbareel	0	0.967	0.960	301.497	Chain A: 45,184,185,186,187,190,213,214,215,217,218,219,221,222,244,245,246,250,254,257,258,276,277,278
site93_6	α12/α13 HTH	0	0.776	0.766	199.283	Chain B: 19,20,21,22,23,41,48,67,69,226,227,230,231,266,269,271,272,273,274,275,276,277
site103_7	HTH1-beta barrel	0	0.786	0.772	160.867	Chain B: 184,185,186,187,188,190,214,217,218,221,245,246
site107_8	Dimerization	0	0.838	0.862	174.930	Chain A: 49,50,51,55,57,111,112,113,115,116,117,118,148,149,151,152,154; Chain B: 132,133,134,137
site122_9	Dimerization	0	0.731	0.709	127.939	Chain A: 131,132,133,134; Chain B: 112,113,115,116,117,118,148,151,152,153,154
site123_10	A12HTH	0	0.674	0.643	110.103	Chain A: 20,21,22,41,43,48,69,272,273,274,275,276,277
site8_11	Orthosteric	100	1.034	0.916	360.836	Chain A: 24,26,27,28,30,44,45,46,47,60,61,62,63,64,107,108,109,170,171,174,175,178,179,181,208,209,210,211,216,219,220,223,224,227,258
site10_12	Interface	100	1.068	1.075	227.066	Chain A: 116,117,119,120,121,123,124,126,127,128,137,141,144; Chain B: 116,117,120,121,123,124,127,128,131,133,137,141,144,148
site19_13	Orthosteric	100	1.059	1.000	324.478	Chain B: 24,26,27,28,42,44,45,46,47,48,49,60,61,62,63,64,104,105,106,107,108,109,110,164,170,174,177,178,181,208,209,211,216,220,223,224
site33_14	Interface	100	0.941	0.974	151.949	Chain A: 92,136,139,140,143,144,147; Chain B: 91,92,137,140,141,143,144,147
site39_15	UNK	100	1.030	1.019	263.081	Chain A: 8,9,10,11,12,28,29,30,58,60,61,78,79,80,81,82,84,85,139,142,161,164,165,168,170,171,174,208
site48_16	α12/α13 HTH	100	0.881	0.758	139.601	Chain B: 22,41,43,45,46,47,48,50,69,258,259,273,274,275,277
site71_17	α12/α13 HTH	100	0.739	0.644	159.495	Chain A: 21,22,24,226,229,230,255,259,263,264,266,267,272,273

site116_19	Interface	100	0.711	0.663	125.881	Chain A: 115,116,117,118,148,151,152,153; Chain B: 127,128,131,132,133
site120_20	Dimerization	100	0.681	0.638	126.567	Chain A: 1; Chain B: 99,100,115,116,117,118,148,150,151,152,153,155,156
site7_41	Orthosteric	400	1.046	0.954	575.211	Chain A: 24,26,27,28,30,44,45,58,60,61,62,63,64,80,81,82,83,85,105,106,107,108,109,161,165,168,171,172,174,175,178,181,208,209,216,219,220,223,224,227,258
site10_42	Interface	400	1.094	0.991	143.374	Chain A: 117,120,121,123,124,125,126,127,128,141,144,148; Chain B: 120,121,123,124,127,128,137,141,144
site19_43	Orthosteric	400	0.977	0.867	227.752	Chain B: 44,45,46,47,49,60,61,62,64,106,107,108,109,174,208,209,210,211,216,220
site35_44	Dimerization	400	0.911	0.945	248.332	Chain A: 8,9,10,11,12,79,80,81,84,91,92,136,138,139,140,142,143,147; Chain B: 91,92,95,136,140,143
site47_45	Orthosteric	400	0.897	0.608	65.856	Chain A: 24,44,45,62,63,64,65,223,258,259,275
site75_47	α12/α13 HTH	400	0.802	0.767	186.249	Chain B: 20,21,22,41,42,43,48,50,67,69,226,230,266,269,271,272,273,274,276,277
site79_48	Dimerization	400	0.792	0.760	71.001	Chain B: 89,93,96,97,99,100,101,159,162,163
site88_49	UNK	400	0.721	0.623	133.427	Chain A: 102,104,105,106,107,157,160,163,164,167,169,170,204,207,208
site92_50	Dimerization	400	0.761	0.718	138.229	Chain A: 10,12,14,16,36,37,74,76,126,129,130,132,133,134,135,138
site8_81	Orthosteric	800	1.072	0.972	602.308	Chain A: 22,24,26,27,28,42,43,44,45,59,60,61,62,63,64,65,108,109,174,177,178,181,209,210,211,216,219,220,223,224,227,230,258,259,272,273,274,275
site10_82	Interface	800	1.124	1.107	281.603	Chain A: 34,78,117,118,120,121,122,123,124,125,127,128,131,133,137,141,144,145,148,149; Chain B: 116,117,120,121,123,124,127,128,137,141,144,148
site19_83	HTHbarel	800	0.932	0.694	153.321	Chain B: 44,45,46,47,49,60,61,62,64,106,107,108,109,174,208,209,210,211,216,220
site55_84	Dimerization	800	0.844	0.779	143.031	Chain A: 92,128,133,137,140,141,143,144; Chain B: 92,140,143,144,147
site56_85	UNK	800	0.787	0.726	173.558	Chain B: 11,29,30,60,61,79,80,81,82,83,84,85,139,142,165,168,171,174
site71_86	α12/α13 HTH	800	0.881	0.837	173.558	Chain A: 21,22,23,24,43,65,226,229,230,259,266,267,269,271,272,273,274,275
site74_87	HTHbareel	800	0.849	0.856	171.500	Chain B: 24,26,27,28,61,62,63,174,177,178,181,182,209,216,220,221,224,242
site81_88	UNK	800	0.789	0.717	153.321	Chain A: 9,10,29,30,61,79,80,81,82,83,84,139,142,165,168,171,174,175
site92_89	Dimerization	800	0.811	0.756	136.171	Chain A: 11,12,13,14,16,36,74,75,76,125,126,129,130,132,133,135,138
site95_90	A12HTH	800	0.772	0.674	90.895	Chain B: 22,43,44,45,46,47,48,65,223,226,258,259,273,275

site12_111	Orthosteric	1100	1.024	0.977	428.750	Chain A: 24,26,27,28,29,30,44,45,46,60,61,62,63,64,80,81,82,83,85,86,107,108,109,161,164,165,167,168,170,171,174,175,178,181,208,209,211,216,220,223,224
site20_112	Interface	1100	1.002	1.055	613.970	Chain A: 2,3,4,5,6,10,112,113,115,117,118,120,121,123,124,127,128,131,132,133,134,135,136,137,140,141,144,148,151,152,153,154; Chain B: 112,113,114,115,116,117,118,128,131,132,133,134,137,140,141,144,147,148,149,151,152,153,154
site21_113	Interface	1100	1.001	1.019	150.920	Chain A: 90,91,92; Chain B: 2,3,4,5,6,8,11,83,84,86,87,90,136,139,143
site31_114	α12/α13 HTH	1100	1.014	1.018	248.675	Chain B: 21,22,23,24,25,43,65,223,226,227,229,230,255,258,259,267,273,276,278
site56_115	Dimerization	1100	1.013	1.048	264.110	Chain B: 6,8,9,10,11,29,30,31,58,60,61,77,79,80,81,82,83,84,85,86,146,149,158,161,164,165,166,167,170,171,174,175
site71_116	α12/α13 HTH	1100	0.943	0.931	185.563	Chain A: 21,22,23,24,226,227,229,230,266,267,269,271,272,273
site72_117	Orthosteric	1100	0.963	0.991	177.674	Chain B: 13,15,17,19,25,26,27,28,29,33,40,42,59,64,66,75,77,79
site88_118	UNK	1100	0.806	0.813	172.186	Chain A: 101,102,103,104,105,106,160,163,164,167,202,203,204,205,206,207,210,211,213
site125_120	HTHbareel	1100	0.667	0.619	86.779	Chain B: 218,219,221,222,245,250,254,257,258
site10_151	Interface	1500	0.911	0.905	341.285	Chain A: 1,117,120,124,127,128,137,140,141,144,148; Chain B: 117,120,121,124,127,128,131,137,141,144,148
site39_152	UNK	1500	1.028	1.093	237.699	Chain A: 28,29,30,58,60,61,79,80,81,82,85,161,162,164,165,166,167,168,170,171,174
site43_153	Dimerization	1500	0.898	0.911	228.438	Chain A: 2,3,4,7; Chain B: 95,101,112,115,116,117,118,148,150,151,152,153,154,155,156,159
site45_154	α12/α13 HTH	1500	0.915	0.922	175.273	Chain A: 20,21,22,41,50,67,69,226,230,266,269,271,272,273,274,275
site48_155	α12/α13 HTH	1500	0.875	0.648	111.818	Chain B: 22,43,45,46,47,48,50,69,258,259,272,273,274,275,276
site56_156	UNK	1500	1.027	1.034	184.877	Chain B: 29,30,58,60,61,80,81,82,83,85,86,88,106,146,149,158,161,162,164,165,167,170,171,174
site67_157	α12/α13 HTH	1500	0.838	0.752	163.611	Chain B: 21,22,226,229,230,248,251,252,255,256,259,260,261,262,263,266,267,278
site104_158	Interface	1500	0.799	0.693	122.451	Chain A: 90,91; Chain B: 1,2,3,4,83,84,85,86,87,88,91,143
site112_159	Interface	1500	0.764	0.744	175.959	Chain A: 94,95,98,99,100,101,117,147,148,150,151,155,156,159; Chain B: 1,7,9,134,135,136,137
site119_160	HTHbareel	1500	0.693	0.652	109.760	Chain A: 181,182,183,184,185,186,187,217,218,221,222,243,244,245,246
site10_211	Interface	2100	1.062	1.080	274.743	Chain A: 116,117,120,121,123,124,127,128,130,131,133,134,137,141,144,148; Chain B: 117,120,121,123,124,127,128,137,141,144,148,151

site12_212	Orthosteric	2100	1.020	1.021	373.527	Chain A: 9,10,13,26,27,29,30,44,45,58,60,61,62,63,64,77,79,80,81,82,83,85,108,109,158,161,162,164,165,167,170,171,174,178,208,209,211,216,220,223,258
site36_213	Dimerization	2100	0.920	0.951	199.283	Chain A: 91,92; Chain B: 11,12,13,76,77,78,79,81,84,87,88,125,135,136,138,139,140,142,143
site56_214	UNK	2100	0.807	0.777	173.558	Chain B: 28,29,30,60,61,79,80,81,82,85,161,164,165,170,171,174,208
site73_217	HTH1-beta barrel	2100	0.650	0.608	193.109	Chain A: 183,184,185,186,187,190,214,217,218,219,221,222,243,244,245,254,257,258,276
site88_218	UNK	2100	0.838	0.859	222.607	Chain A: 101,102,104,105,106,160,163,164,167,169,170,196,202,203,204,205,206,207,210,211,212,213
site103_219	HTH1-beta barrel	2100	0.712	0.622	152.635	Chain B: 181,183,184,185,186,187,188,190,214,217,218,221,243,244,245
site8_241	HTH1-beta barrel	2400	1.078	0.887	335.797	Chain A: 24,26,27,28,44,45,60,61,62,63,64,107,108,109,174,177,178,181,182,206,207,208,209,210,211,216,217,220,221,224,242
site10_242	Interface	2400	1.051	1.078	385.532	Chain A: 117,120,121,123,124,127,128,131,133,141,144,148,151; Chain B: 117,120,121,124,127,128,131,133,134,137,141,144,148
site32_243	Interface	2400	0.860	0.868	267.883	Chain A: 84,87,91,92,136,139,143; Chain B: 84,87,88,90,91,94,139,140,143,147
site39_244	UNK	2400	0.794	0.743	147.490	Chain A: 28,29,30,60,61,80,81,82,85,86,165,166,167,168,170,171,174,208
site56_245	Dimerization	2400	0.856	0.842	174.930	Chain B: 29,30,58,60,61,80,81,82,85,161,164,165,170,171,174,175
site69_246	HTH1-beta barrel	2400	0.862	0.771	247.303	Chain B: 43,45,46,65,181,183,184,185,186,187,190,214,217,218,219,221,222,243,244,245,250,253,254,257,258,276
site85_247	Dimerization	2400	0.786	0.789	216.090	Chain B: 11,12,13,14,15,17,25,26,27,28,29,33,75,76,77,78,79,84,125,133,135,138,139,142
site97_248	Dimerization	2400	0.764	0.722	165.326	Chain A: 49,106,107,110,111,112,113,115,116,117,118,148,151,152,153,154,157; Chain B: 1
site8_281	Orthosteric	2800	0.999	0.937	352.604	Chain A: 24,26,28,44,45,46,47,49,60,61,62,63,64,101,103,105,106,107,108,154,157,174,177,178,180,181,208,209,211,212,215,216,219,220,224,227,228
site10_282	Interface	2800	1.059	0.992	379.015	Chain A: 117,120,121,123,124,127,128,131,133,137,138,140,141,144,148; Chain B: 117,120,121,124,127,128,131,137,140,141,144,147,148
site25_283	ALL	2800	0.992	1.017	740.194	Chain A: 5,84,87,88,90,91,92,93,94,136,139,140,143; Chain B: 11,12,13,14,15,17,25,26,27,28,29,30,33,58,60,61,66,75,76,77,79,80,81,82,83,84,85,86,87,88,90,91,135,136,139,142,143,147,161,164,165,167,168,170,171,174,175,208
site39_284	Dimerization	2800	1.070	1.091	292.236	Chain A: 5,6,7,9,10,11,12,28,29,30,60,61,78,79,80,81,82,84,85,86,125,128,138,139,142,165,168,170,171,174,175
site89_287	Orthosteric	2800	0.829	0.836	110.789	Chain A: 17,19,25,26,27,28,29,33,42,57,59,64,66,77,109

site107_289	Dimerization	2800	0.718	0.694	144.403	Chain A: 49,50,51,55,111,112,113,115,116,117,118,148,151,152,153,154
site8_301	Orthosteric	3000	0.981	0.950	566.979	Chain A: 24,26,27,44,45,46,47,49,60,61,62,63,64,103,104,105,106,107,108,109,110,111,154,157,160,163,164,170,174,177,178,181,206,207,208,209,210,211,212,215,216,219,220,224,227
site10_302	Interface	3000	1.090	1.116	352.947	Chain A: 117,120,121,123,124,127,128,131,133,137,138,140,141,144,148; Chain B: 117,120,121,124,125,127,128,137,141,144,145
site32_303	Interface	3000	1.030	1.000	339.227	Chain A: 2,3,5,6,11,84,87,88,90,91,92,136,139,140,143; Chain B: 87,88,90,91,94,136,139,140,143,147
site39_304	Dimerization	3000	0.917	0.891	139.601	Chain A: 30,58,60,61,80,81,82,85,146,158,161,162,164,165,170,171,174,175
site72_306	Orthosteric	3000	0.888	0.919	139.944	Chain B: 17,19,25,26,27,28,29,33,40,42,59,64,66,77
site105_308	UNK	3000	0.811	0.831	215.061	Chain B: 60,105,106,107,108,109,164,167,170,200,203,204,206,207,208,210
site107_309	Dimerization	3000	0.705	0.668	119.021	Chain A: 112,113,115,116,117,118,148,151,152,153,154; Chain B: 1,2
site122_310	Dimerization	3000	0.705	0.679	93.982	Chain B: 112,113,115,116,117,118,148,149,151,152,153,154

References

(1) Schrödinger LLC, N. Y., NY (2024) Schrödinger Software Suite. Release 2024-3. SiteMap.
(2) Halgren, T. A. Identifying and Characterizing Binding Sites and Assessing Druggability. *J. Chem. Inf. Model.* **2009**, *49* (2), 377–389. https://doi.org/10.1021/ci800324m.
(3) Loving, K. A.; Lin, A.; Cheng, A. C. Structure-based druggability assessment of the mammalian structural proteome with inclusion of light protein flexibility. *PLoS Comput. Biol.* **2014**, *10*, e1003741.
(4) Kumar, A.; Kaynak, B. T.; Dorman, K. S.; Doruker, P.; Jernigan, R. L. Predicting allosteric pockets in protein biological assemblages. *Bioinformatics* **2023**, *39*, btad275.
(5) Le Guilloux, V.; Schmidtke, P.; Tuffery, P. Fpocket: An open source platform for ligand pocket detection. *BMC Bioinform* **2009**, *10*, 168.
(6) Tian, H.; Xiao, S.; Jiang, X.; Tao, P. PASSer: fast and accurate prediction of protein allosteric sites. *Nucleic Acids Res.* **2023**, *51*, W427–W431.
(7) Krapp, L. F.; Abriata, L. A.; Cortés Rodriguez, F.; Dal Peraro, M. PeSTo: parameter-free geometric deep learning for accurate prediction of protein binding interfaces. *Nat. Commun.* **2023**, *14*, 2175.
